# Peripheral Delivery of a CNS Targeted, Metalo-Protease Reduces Aβ Toxicity in a Mouse Model of Alzheimer's Disease

**DOI:** 10.1371/journal.pone.0016575

**Published:** 2011-01-31

**Authors:** Brian Spencer, Robert A. Marr, Ryan Gindi, Rewati Potkar, Sarah Michael, Anthony Adame, Edward Rockenstein, Inder M. Verma, Eliezer Masliah

**Affiliations:** 1 Department of Neurosciences, University of California San Diego, La Jolla, California, United States of America; 2 Department of Neuroscience, Center for Stem Cell and Regenerative Medicine, Rosalind Franklin University of Medicine and Science, North Chicago, Illinois, United States of America; 3 Laboratory of Genetics, Salk Institute for Biological Studies, San Diego, California, United States of America; 4 Department of Pathology, University of California San Diego, La Jolla, California, United States of America; Mental Health Research Institute of Victoria, Australia

## Abstract

Alzheimer's disease (AD), an incurable, progressive neurodegenerative disorder, is the most common form of dementia. Therapeutic options have been elusive due to the inability to deliver proteins across the blood-brain barrier (BBB). In order to improve the therapeutic potential for AD, we utilized a promising new approach for delivery of proteins across the BBB. We generated a lentivirus vector expressing the amyloid β-degrading enzyme, neprilysin, fused to the ApoB transport domain and delivered this by intra-peritoneal injection to amyloid protein precursor (APP) transgenic model of AD. Treated mice had reduced levels of Aβ, reduced plaques and increased synaptic density in the CNS. Furthermore, mice treated with the neprilysin targeting the CNS had a reversal of memory deficits. Thus, the addition of the ApoB transport domain to the secreted neprilysin generated a non-invasive therapeutic approach that may be a potential treatment in patients with AD.

## Introduction

Alzheimer's disease (AD) is an incurable progressive neurodegenerative disorder affecting over 10 million people in the US alone[1]. This neurological disorder is characterized by widespread neurodegeneration throughout the association cortex and limbic system, deposition of Aβ in the neuropil and around the blood vessels, and formation of neurofibrillary tangles[2]. In spite of the considerable progress towards better understanding the pathogenesis of AD, no effective therapeutic approaches are currently available. A fundamental problem toward the goal of developing new therapies for AD has been the difficulty in crossing the blood brain barrier (BBB)[3].

Experimental treatments for AD include reducing the synthesis or aggregation of Aβ or increasing the clearance of Aβ. Recently, progress has been made towards identifying endopeptidases, which directly degrade Aβ and play an important role in the homeostatic control of this peptide. Among them, Neprilysin (NEP, also known as CD10, EC 3.4.24.11)—a zinc metalloendopeptidase—has been identified as a critical Aβ-degrading enzyme in the brain[4], [5], [6]. Neprilysin has been shown to degrade Aβ monomers; however, the ability of NEP to degrade Aβ-oligomers is controversial while some groups have reported that this endopeptidase breaks down oligomers[7], [8], others have not seen such effects[9], [10]. Neprilysin levels are reduced in the brains of AD patients and a potential genetic linkage is currently being investigated (reviewed in[11]).

We and other groups have shown that overexpression of NEP by gene transfer with viral vectors[12], [13], [14], transgenesis[15], or induction[16], [17], [18], [19], [20] resulted in a reduction in amyloid pathology.

Viral vector gene delivery of NEP via stereotactic injection into the CNS has proven to be a viable approach to treating the small brain of mice or rats; however, scaling up to the size of the human brain would require numerous injections that would make these treatments undesirable. An alternative approach for delivery of a therapeutic protein to the CNS is by transport across the BBB.

Recently, a novel approach was developed for delivering therapeutic proteins to neurons of the CNS by targeting passage across the BBB[21]. Fusion of the Apolipoprotein B (ApoB) low-density lipoprotein (LDL) receptor-binding domain to a targeted protein allows active transport of the protein across the BBB to the CNS. The fusion proteins can be taken up by neurons and astrocytes across the whole brain. To investigate the potential therapeutic value of a secreted NEP targeted to the CNS, we generated lentivirus vectors expressing either the wildtype NEP, a secreted form of the NEP or a secreted form of the NEP fused with the LDL-receptor binding domain of ApoB and injected them intra-peritoneally in an amyloid protein precursor (APP) transgenic (tg) model of AD-like pathology. We found that ApoBSecNEP was efficiently trafficked into the CNS and reduced levels of Aβ and synaptic alterations in the brains of mice. In addition, we observed improvements in learning and memory just 1 month after vector delivery. These results suggest a novel, and improved approach for delivery of an Aβ degrading enzyme and other neuroactive peptides to the CNS for treatment of AD.

## Results

### The apoB-secreted neprilysin fusion protein is active and functions similarly to endogenous neprilysin

To determine if the fusion ApoBSecNEP protein and SecNEP variant proteins were secreted and active, lentiviruses were produced and used to infect 293T cells. Infected cell lysates and conditioned media were collected 72 hours after virus infection and analyzed by western blot. The NEP antibody recognized a band at ∼100 kDa in the lysates of the 293T cells infected with the LV-NEP, LV-SecNEP and the LV-ApoBSecNEP (Figure S1B). This band was not observed in uninfected 293T cells or cells infected with the LV-ApoBGFP virus indicating that NEP is not endogenously produced by the 293T cells. Conditioned media contained the secreted NEP reacting band at 100 kDa only in cultures infected with the LV-SecNEP and LV-ApoBSecNEP viruses whereas the media from control cells or cells infected with the LV-NEP did not contain NEP (Figure S1C).

**Figure 1:**
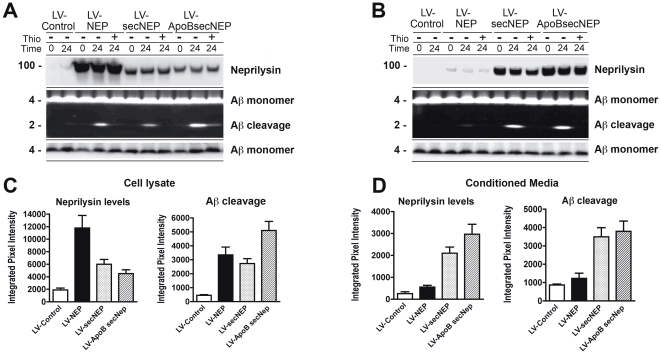
Characterization of levels of expression and activity of the ApoBSecNEP fusion protein. 293T cells were infected with LV-NEP, LV-SecNEP or LV-ApoBSecNEP and 48 hours later analyzed. (A) Representative images of immunoblot of cell lysates probed with anti-Neprilysin (upper panel) and anti-Aβ (lower panel) antibodies. The middle panel illustrates the proteolytic activity against a FITC labeled Aβ_42_. (B) Representative images of immunoblot and proteolysis of FITC labeled Aβ_42_ with conditioned media. Reactions were collected at 0 or 24 hours in the presence or absence of the neprilysin inhibitor thiorphan and analyzed with the Versadoc gel imaging system (BioRad). (C) Analysis of levels of neprilysin immunoreactive bands and proteolysis of FITC labeled Aβ_42_ with cell lysates. (D) Levels of neprilysin immunoreactivity and proteolysis of FITC labeled Aβ_42_ with conditioned media.

In order to determine if the vectors expressing the fusion NEP constructs were enzymatically active, cell lysates or conditioned media samples were incubated with an N-terminus FITC labeled Aβ_42_ for 24 hours. In this system, NEP activity is detected by the presence of a 2 kDa band corresponding to the N-terminal cleavage product of Aβ. Lysates of infected 293T cells showed increased NEP activity from all the NEP vectors tested with the wildtype NEP displaying a 6 fold increase in Aβ cleavage compared to uninfected 293T cells (Figure 1A). Of particular interest, the SecNEP and ApoBSecNEP proteins were present at reduced levels in the lysates of the infected cells, however the NEP activity was as efficient as wild type NEP with the SecNEP and 50% higher with the ApoBSecNEP (Figure 1C)

Conditioned media from infected cells was subjected to the same N-terminus FITC labeled Aβ cleavage assay (Figure 1B). Low levels of Aβ cleavage activity were observed from uninfected and LV-NEP infected conditioned media, however as expected, NEP activity was present in the conditioned media of cells infected with either the LV-SecNEP or LV-ApoBSecNEP vectors. Aβ cleavage was detected at levels comparable to cell lysates only in those samples that had the secreted NEP (Figure 1D). The Aβ cleavage was specific to the presence of NEP as the addition of thiorphan, a NEP specific inhibitor, blocked the formation of the Aβ cleavage product (Figure 1A,B).

**Figure 2:**
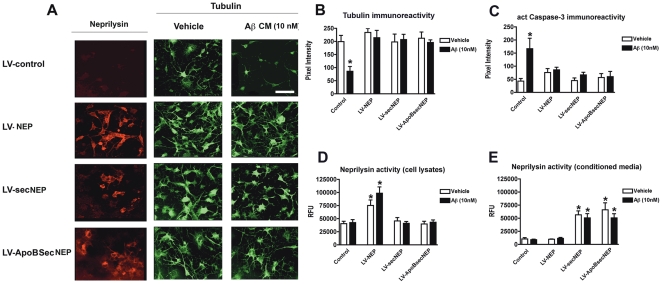
Neuroprotective effects of the ApoBSecNEP fusion protein from Aβ toxicity. Adult rat hippocampal neural progenitor cells were differentiated into neurons, infected with LV-control, LV-NEP, LV-SecNEP or LV-ApoBSecNEP for 48 hours and then challenged with 10 nM Aβ for 24 hours. Neuronal cells were immunostained and analyzed by confocal laser microscopy. (A) Patterns of immunostaining with antibodies against neprilysin (first column). Comparison of effects of Aβ on neuronal morphology and neurite characteristics with an antibody against β-tubulin (second and third columns). Scale bar  = 50 µm. (B) Computer aided image analysis of the levels of β-tubulin immunoreactivity in cells infected with the lentiviruses and treated with vehicle alone or Aβ. (C) Levels of activated caspase-3 immunoreactivity (marker of cell injury via pro-apoptotic pathway) determined by immunocytochemistry and computer aided image analysis in cells infected with the lentiviruses and treated with vehicle alone or Aβ. (D and E) Determinations of levels of neprilysin activity in cell lysates and the conditioned media utilizing an artificial substrate (DAGNPG). * - indicates statistically significant difference by 1-way ANOVA with poshoc Dunnet's when compared to vehicle treated control (p<0.05).

To determine if the fusion NEP constructs were active in a cell based system, differentiated adult rat neural progenitor cells (NPCs) exposed to Aβ were utilized. In this system, differentiated NPCs treated for 24 hours with Aβ showed reduced levels of β-tubulin immunoreactivity and increased activated caspase 3 levels indicating Aβ induced toxicity (Figure 2A–C). To examine whether the recombinant NEP could protect the differentiated NPCs cells from the Aβ induced toxicity, cells were infected with the LV-NEP, LV-SecNEP or LV-ApoBSecNEP and then challenged with Aβ (10 nM) for 24 hours. Cells that expressed either the wildtype NEP or either of the secreted NEP proteins had increased β-tubulin immunoreactivity and showed no increase in activated caspase 3 (Figure 2A–C). Thus, the ApoBSecNEP fusion protein is capable of protecting the NPC cells *in vitro*.

In conclusion, the lentiviral vectors containing SecNEP and fusion ApoBSecNEP express the NEP protein and secrete the protein into the supernatent of infected cells. In addition, the fusion NEP constructs are active at cleaving the Aβ_42_ protein in a similar manner to the wildtype NEP and can protect differentiated NPCs from an extracellular challenge of Aβ. The next step was to determine if the ApoBSecNEP protein traffics into the brain of APP tg mice and if it reduces the pathology and deficits associated with amyloid production.

### ApoBSecNEP traffics into the CNS of APP tg mice and accumulates in neurons in the hipoocampus and neocortex

**Figure 3:**
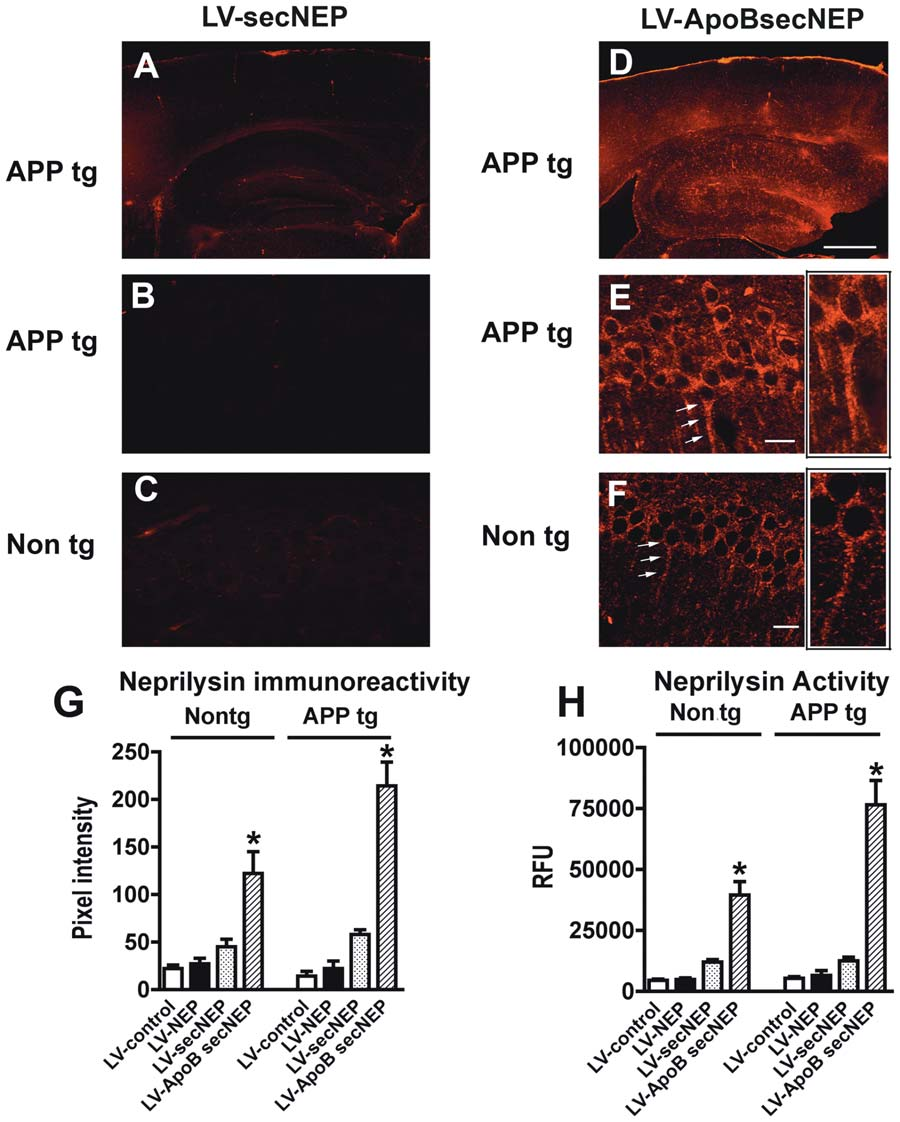
Trafficking of the ApoBSecNEP into the CNS of APP tg mice. Vibratome sections from nontg and APP tg mice that received a single intra-peritoneal injection of LV-control, LV-NEP, LV-SecNEP or LV-ApoBSecNEP were immunostained with an antibody against NEP and analyzed by laser scanning confocal microscopy. (A, B) Low levels of NEP immunostaining in APP tg mice and (C) nontg mice treated with LV-secNEP (D, E) Accumulation of NEP immunoreactivity (red) in the neocortex and hippocampus of APP tg mice and (F) to a lower extent in nontg mice treated with LV-ApoBsecNEP. Areas of neprilysin immunolabeling (white arrows) were enlarged to show localization within neurons (cutouts E, F). Scale bar  = 100 µm for A, B and 20 µm for C–F. (G) Computer aided image analysis of levels of neprilysin immunoreactivity expressed as pixel intensity. (H) Determinations of levels of neprilysin activity in hippocampal homogenates by measuring an artificial substrate cleavage (DAGNPG). * - indicates statistically significant difference by 1-way ANOVA with poshoc Dunnet's when compared to mice treated with the LV-Control (p<0.05). n = 8 mice per group.

To determine whether these vectors might be effective at reducing the levels of Aβ *in vivo*, we delivered 1×10^9^ tdu of lentivirus vector to 6 month old APP tg mice via a single intra-peritoneal injection. One month after injection, mice were sacrificed and brains were removed for analysis of Aβ and NEP. In order to verify that the ApoBSecNEP molecule trafficked into the CNS, immunohistochemical analysis was performed. One month after lentivirus delivery in the periphery, widespread NEP uptake was detected only in those mice that had received the LV-ApoBSecNEP injections (Figure 3D-F) whereas only low levels of NEP were observed in those mice that received LV-SecNep (Figure 3A–C). Neprilysin was particularly concentrated in the hippocampus and the lower layers of the cortex and could be identified in both the soma and axon of neurons. Consistent with these results and our previous studies[21], control experiments utilizing a lentiviral vector expressing the ApoB-GFP fusion protein confirmed that at 30 days after intra-peritoneal injections, GFP accumulation was detected in the hippocampus (Figure S2).

Analysis of the levels of NEP immunoreactivity (Figure 3G) and enzyme activity (Figure 3H) showed almost 10 fold more enzyme in the brains of mice that had received the ApoBSecNEP compared to those that received SecNEP or control animals. Interestingly, the levels of NEP enzyme that accumulated in the CNS of the APP tg mice was significantly greater than in the brains of nontg mice. Western blot analysis from the CNS confirmed the immunohistochemical results (Figure S3).

**Figure 4:**
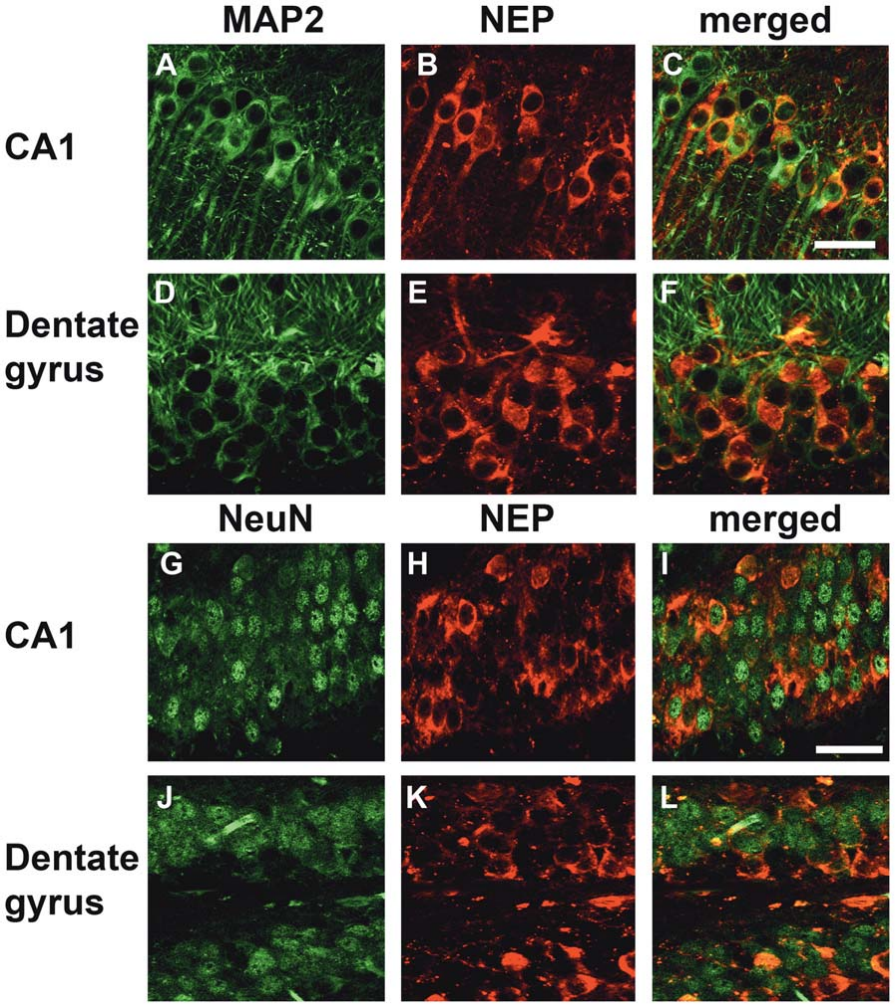
Localization of ApoBSecNEP in hippocampus of APP tg mice. Sections from APP tg mice that received a single intraperitoneal injection of LV-ApoBSecNEP were double labeled with antibodies against NEP (panels in red) and neuronal markers MAP2 or NeuN (panels in green). (A–C) Co-localization of NEP with MAP2 in the pyramidal neurons in the CA1 regions of the hippocampus; (D–F) co-localization of NEP with MAP2 in granular neurons in the dentate gyrus. (G–I) Co-localization of NEP with NeuN in the pyramidal neurons in the CA1 regions of the hippocampus; (J–L) co-localization of NEP with NeuN in granular neurons in the dentate gyrus. Scale bar  = 50 µm.

Closer examination of the sections from mice that received the LV-ApoBSecNEP showed recombinant protein throughout the hippocampus. In particular, NEP co-localized with NeuN and MAP2 positive neurons in the CA1 region and dentate gyrus of the hippocampus (Figure 4). Intracellular recombinant protein accumulated in the neuronal soma with some extending into the axons (Figures 3E, 4A–C).

### ApoBSecNEP reduces the levels of Aβ in APP tg mice

**Figure 5:**
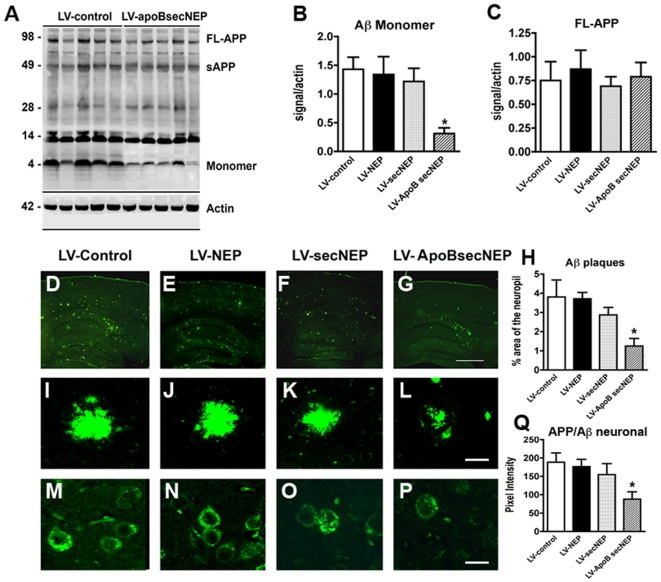
ApoBSecNEP reduces Aβ in the CNS of APP tg mice. Sections from APP tg mice that had received peripheral injections with the lentiviral vectors were homogenized, fractioned and examined for levels of APP and Aβ by western blot. (A) Representative immunoblot probed with the 82E1 monoclonal antibody displaying the reduction in the levels of Aβ in APP tg mice treated with LV-ApoBSecNEP. (B–C) Analysis of immunoblot for levels of Aβ monomers and full length APP. (D–G) Low magnification view of sections from APP tg mice treated with LV-control, LV-NEP, LV-SecNEP or LV-ApoBSecNEP respectively and immunolabeled with a monoclonal antibody against Aβ (82E1) imaged with the laser scanning microscope. (H) Computer aided image analysis of the % area of the neuropil occupied by Aβ immunoreactive deposits. (I–L) Higher magnification view of the Aβ immunoreactive plaques in APP tg mice treated with the various lentiviruses. (M–P) Representative images of the patterns of intraneuronal APP/Aβ immunostaining in the frontal cortex from APP tg mice treated with LV-Control, LV-NEP, LV-SecNEP or LV-ApoBSecNEP respectively immunolabeled with a monoclonal antibody against Aβ (82E1) imaged with the laser scanning microscope. (Q) Image analysis of levels of intracellular APP/Aβ immunostaining. Scale bar  = 15 µm for I-L and 10 µm for M-P. * - indicates statistically significant difference by 1-way ANOVA with poshoc Dunnet's when compared to LV-Control treated animals (p<0.05). n = 8 mice per group.

Previous studies have shown that NEP preferentially degrades monomeric forms of Aβ rather than fibrils[9], [10]. Thus to evaluate the effects of ApoBSecNEP on Aβ, we dissected the hippocampus of the mice treated with the lentiviral vectors and analyzed by immunoblot. Compared to APP tg mice treated with LV-control, LV-NEP or LV-secNEP, treatment with LV-ApoBSecNEP resulted in a 60% reduction in the levels of Aβ monomer (Figure 5A,B). In addition, the levels of an Aβ immunoreactive band right above the 4 kDa monomer was noted to be significantly reduced with the LV-ApoBSecNEP. The identity of this band at approximately 6 kDa is unclear but might represent a post-transcriptionally modified version of Aβ that is sensitive to NEP proteolysis. In contrast, no significant effects were observed in the levels of full length APP (Figure 5A,C).

When the effects of the LV treatments were analyzed by immunocytochemistry, one month followed intra-peritoneal delivery of the LV-ApoBSecNEP, mice had a 70% reduction in the load of Aβ immunoreactive plaques in the neocortex and hippocampus compared to APP tg mice treated with either LV-control and LV-NEP (Figure 5D–H; 5I–L). The APP tg mice treated with LV-secNEP displayed a 20% reduction in the load of Aβ immunoreactive plaques in the hippocampus (Figure 5H).

**Figure 6:**
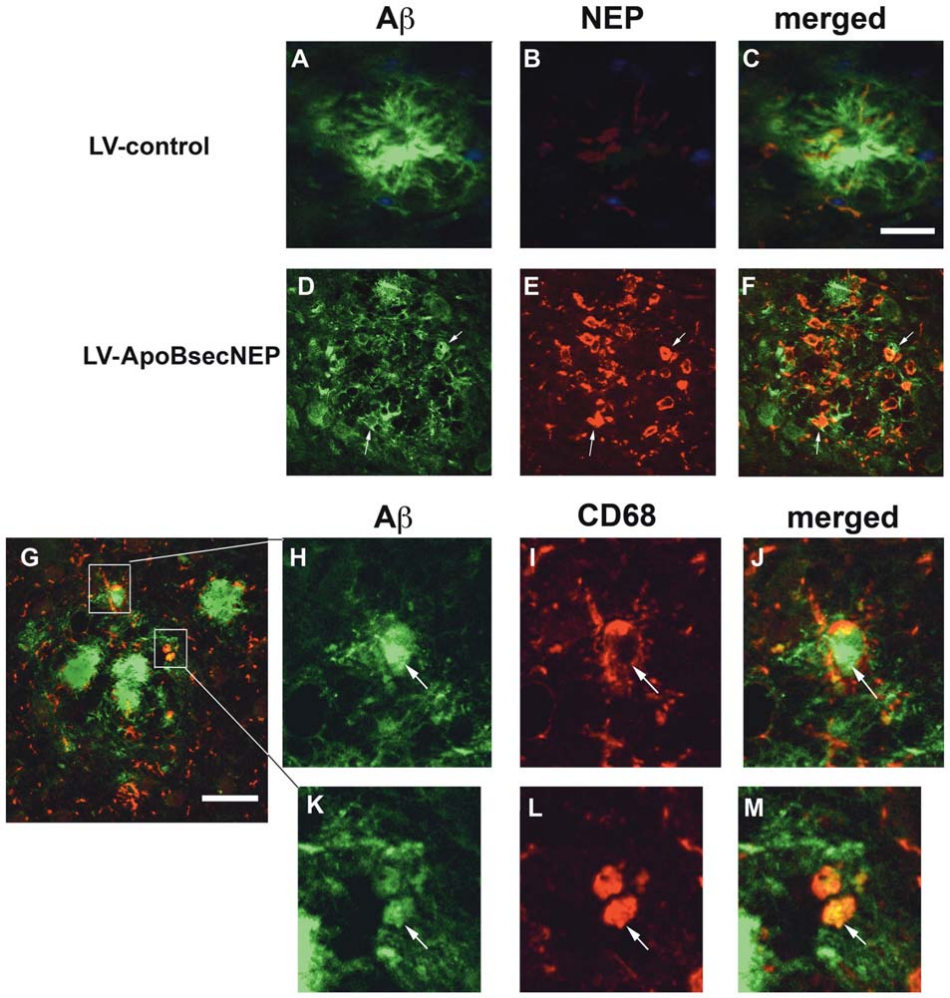
ApoBSecNEP co-localizes with activated macrophages at the site of plaques. Sections from APP tg mice that received peripheral injections with (A–C) LV-control or (D–F) LV-ApoBSecNEP were double labeled with antibodies against Aβ (green) and NEP (red) and imaged with the laser scanning confocal microscope. (G–M) Representative plaque from a mouse treated with LV-ApoBSecNEP displaying co-localization of Aβ immunoreactive material in macrophages/microglia around the plaques labeled with CD68 (red). White arrows indicate areas of co-localization of Aβ and neprilysin or CD68. Scale bar  = 20 µm for A–F. Scale bar  = 10 µm for G–M.

Since NEP favors the cleavage of monomers rather than fibrils, the reduction in the number of plaques might be driven in part by the change in ratio between monomeric and aggregated Aβ, however other factors might be involved including increased clearing by microglia. Consistent with this possibility, double labeling and confocal microscopy showed that compared to APP tg mice treated with LV-control, in mice treated with LV-ApoBsecNEP macrophage/microglial cells embedded in the plaques displayed abundant NEP immunoreactivity (Figure 6). Higher resolution and double labeling analysis confirmed that the cells associated with the plaques were immmunostained with antibodies against macrophage markers such as CD68 and CD11b and contained abundant aggregated Aβ immunoreactive material (Figure S4). Taken together, these results suggest that the ApoBSecNEP might also be taken up by macrophages and activate these cells to promote amyloid clearance.

In addition to the effects on extracellular Aβ, NEP might modify AD-like pathology by reducing the accumulation of intracellular APP metabolites such as Aβ[22], [23]. Increasingly, intracellular Aβ is being recognized as deleterious to the neuron as much or more than extracellular Aβ[23], [24], [25]. Detection of intracellular Aβ by immunocytochemistry is difficult because of the cross-reactivity of most antibodies with APP. However, some studies have suggested that use of antibodies against the amino terminal of Aβ might be helpful [23], [24], [25]. Immunocytochemical analysis with the monoclonal antibody against Aβ 1-16 (clone 82E1) showed that compared to APP tg mice treated with LV-control, LV-NEP or LV-secNEP, mice treated with LV-ApoBSecNEP displayed a 60% reduction in the levels of intracellular APP/Aβ immunoreactivity (Figure 5M–Q).

### ApoBSecNEP ameliorates the alterations in synaptic markers in APP tg mice

**Figure 7:**
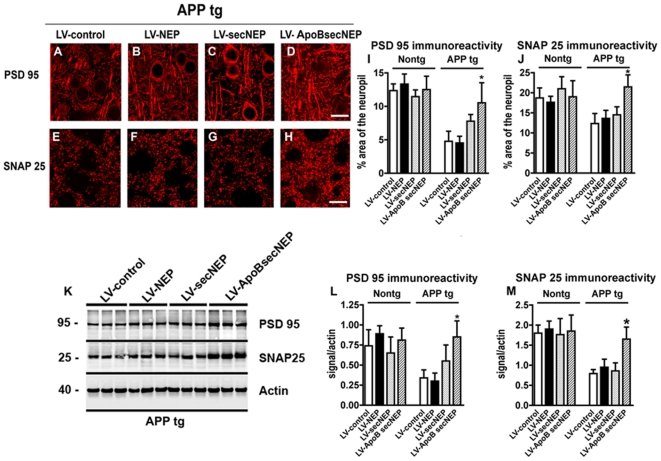
Trafficking of ApoBSecNEP to the CNS ameliorates synaptic deficits in APP tg mice. Brain sections from APP tg mice that had received the lentiviral vectors were immunolabeled with monoclonal antibodies against the post-synaptic marker PSD 95 (red) or the pre-synaptic marker SNAP25 (red) and imaged with the laser scanning confocal microscope. Images are from the frontal-parietal cortex layers 2–3. (A–D) Representative images from APP tg mice treated with LV-control, LV-NEP, LV-SecNEP or LV-ApoBSecNEP respectively and immunolabeled with a monoclonal antibody against PSD 95. (E–H) Images from APP tg mice treated with LV-control, LV-NEP, LV-SecNEP or LV-ApoBSecNEP respectively and immunolabeled with a monoclonal antibody against SNAP25. (I–J) Computer aided image analysis of the % area of the neuropil stained for PSD 95 or SNAP 25 structures. (K) Representative immunoblot probed with antibodies against PSD 95, SNAP25 and actin. For these analysis the posterior aspect of the brain containing the cortex and hippocampus was homogenized and the membrane fraction used for the immunoblot. (L–M) Image analysis of the immunoreactive bands for PSD 95 and SNAP25 expressed as ratio to actin as loading control. Scale bar  = 5 µm for A–H. *  =  indicates statistically significant difference by 1-way ANOVA with poshoc Dunnet's when compared to LV-control treated animals (p<0.05). n = 8 mice per group.

It has been proposed that Aβ neurotoxicity targets the synapses[7], [26], [27]. To determine if delivery of the ApoBSecNEP could correct the synaptic alterations in these mice, immunohistochemical and western blot analysis were performed. Mice that received the LV-control, LV-NEP or LV-SecNEP had significantly decreased levels of the post-synaptic marker, PSD 95, and the pre-synaptic marker, SNAP25, comparable to untreated APP tg mice (Figure 7A–C, E–G). Whereas mice that received the LV-ApoBSecNEP showed levels of both synaptic proteins comparable to nontg mice (Figure 7D,H). Similarly, delivery of the ApoBSecNEP to nontg mice did not appear to affect synaptic protein levels indicating that accumulation of the soluble neprilysin did not have a detrimental effect (Figure 7I,J). Consistent with the immunocytochemical analysis, western blot analysis confirmed that, compared to APP tg mice that received the LV-control, LV-NEP or LV-SecNEP, mice that were treated with LV-ApoBSecNEP displayed levels of PSD 95 and SNAP25 comparable to nontg controls (Figure 7K–M).

### ApoBSecNEP reverse memory deficits in APP tg mice

**Figure 8:**
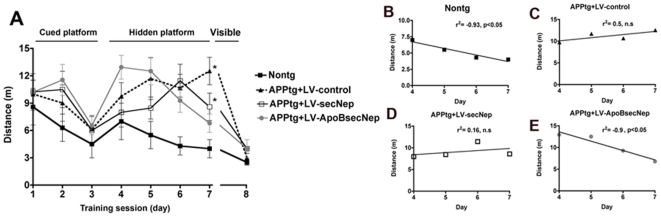
Effects of peripheral treatment with LV-ApoBSecNEP in water maze performance in APP tg mice. (A) Four weeks after the intra-peritoneal injections with the LV-control, LV-SecNEP or LV-ApoBSecNEP virus, memory and learning were assessed by the Morris water maze. Mice were trained on the cued platform on days 1–3 and then tested for spatial learning on days 4–7 followed by a return of the cued platform on day 8. (B–E) Linear regression analysis showing the slope for the learning curves in the nontg mice and the APP tg mice with LV-control, LV-secNEP and LV-ApoB-sec-NEP respectively. *  =  indicates statistically significant difference by 1-way ANOVA with poshoc Dunnet's when compared to nontg treated animals (p<0.05). n = 8 mice per group.

The alterations in synaptic markers in APP tg mice have been associated with memory and learning deficits beginning at 3–6 months of age as measured in the Morris water maze[28]. To determine if delivery of the ApoBSecNEP could ameliorate these deficits, APP tg mice were tested one month after intraperitoneal delivery of either the LV-control, LV-SecNEP or LV-ApoBSecNEP vectors. During the training period of the test, all groups of mice performed similarly at locating the visible platform after 3 days of testing. In the subsequent days of testing (d4-7) with the platform submerged nontg mice located the platform in the water pool, with progressively shorter path distances over time (Figure 8A). Consistently, linear regression analysis showed a significant negative slope when plotting distance over time (Figure 8B). Compared to nontg controls, APP tg mice that received the LV-control did not show improvements in the spatial learning with the submerged platform and the path distance in fact increased over time (Figure 8A) with a positive slope for the linear regression analysis (Figure 8C). Similarly, the APP tg treated with LV-secNEP displayed poor performance taking longer distances to locate the hidden platform for days 4–6, however at day 7 displayed a trend toward an improvement (Figure 8A). However, the linear regression analysis was not significant (Figure 8D). In contrast, APP tg mice that received the LV-ApoBSecNEP vector were able to learn the location of the hidden platform significantly faster and at a rate similar to nontg mice (Figure 8A). In concordance, linear regression analysis showed a significant negative slope when plotting distance over time (Figure 8E). Taken together, these results suggest that delivery of the ApoBSecNEP protein to the CNS of APP tg mice has the capability of ameliorating the synaptic and memory deficits

## Discussion

Development of new approaches for the delivery of neuroactive peptides and compounds across the BBB is of fundamental importance for advancing the therapeutics of AD. For the present study, we showed that the ApoBSecNEP fusion protein was efficiently produced from the lentiviral vector in peripheral organs, trafficked into the CNS and reduced levels of Aβ and synaptic alterations in the brains of mice. In contrast to previous methods for delivery of soluble NEP[29], [30], this study utilized a BBB targeting peptide fused to the soluble protein for transport to the CNS. We had previously shown that we could deliver a recombinant protein across the CNS through the use of the LDL-R binding domain of ApoB[21], however, this is the first report showing that this approach can be used in a therapeutic manner to treat a neurodegenerative disease.

Utilizing this unique approach, intra-peritoneal delivery of the lentivirus vector results in primarily transduction of the liver and spleen[21], [31], with the liver acting as a depot organ for the production and secretion of the recombinant protein. In this study, the ApoBSecNEP expressed in the liver was delivered to the blood stream from where it traffics to the CNS. The ApoB LDL-R binding domain on the recombinant protein specifically facilitates transport across the BBB since the SecNEP protein (lacking apoB) was not present in the CNS following similar vector delivery and did not mitigated the neuropathogy in the APP tg mice. Delivery to the CNS by this route resulted in widespread uptake as evidenced by the immunoreactivity across areas of the CNS including the hippocampus and the neocortex, that are prominently affected in AD[2], [32]. Moreover, our studies showed that the fusion protein displayed, both in vitro and in vivo enzymatic activity comparable to the wildtype secNEP.

This activity included cleavage of an artificial specific substrate, an FITC-tagged Aβ 1-42 and the human Aβ produced in the brains from the transgene. Upon examination of the Aβ species in the CNS, we observed a significant reduction in the monomer Aβ but not full length APP. This is consistent with previous studies showing that NEP favors the degradation of monomeric Aβ over fibrillar species. The effects of the ApoBsecNEP vector on the levels of Aβ oligomers were difficult to determine by western blot given the proximity of the molecular weight with C-terminus fragments of APP. Future studies utilizing SELDI-TOF mass spectrometry analysis will be needed to identify with greater precision trimers and other Aβ multimers in tissues as has been recently done by Crouch et al[33]. However, since the synaptic pathology in AD and in APP tg mice has been associated with the progressive accumulation of Aβ oligomers[34], [35], [36], [37], [38], then it is remarkable that we observed a recovery in the synaptic and behavioral deficits in the APP tg mice treated with ApoBsecNEP. One possible explanation is that even through the levels of existing oligomers were not affected, because of the reduction in the monomers, the progressive generation of more oligomers was decreased or it is also possible that reducing excessive levels of Aβ 1–42 might be beneficial. For example, some studies have shown that in contrast to Aβ 1–40, monomeric Aβ 1–42 could reduce synaptic function and LTP via glutamate receptors[39], [40], [41]. An alternative possibility as shown in this study is that the beneficial effects of ApoBsecNEP might be associated with a decrease in intra-cellular Aβ. A growing number of studies have shown that accumulation of intracellular Aβ could be deleterious[23], [24], [25], [42], [43] by triggering aberrant signaling via CREB[44]. Supporting this possibility, previous studies have shown that NEP ameliorates the deficits in the fly[22] and rodent models[23] by diminishing Aβ from the intracellular compartment. Although ApoBsecNEP was located in the soma and dendrites of neurons, it is not clear if the reduced intracellular Aβ may be driven by this source of NEP. It is also plausible that extracellular ApoBsecNEP is capable of degrading Aβ and this shifts the equilibrium of Aβ from the intracellular space to the extracellular space thus reducing the intracellular accumulation of Aβ indirectly.

In addition to the effects on monomeric and intracellular Aβ, we observed that of ApoBsecNEP trafficking into the CNS reduced the accumulation of amyloid plaques in the APPtg mice. Given that NEP does not degrade Aβ fibrils alternative possibilities were investigated. Remarkably, the present study showed that ApoBsecNEP is also taken up by macrophages/microglia around the plaques and that these cells contained Aβ immunoreactive material, suggesting that NEP activates the macrophages/microglia to clear the Aβ in the plaques. It's not clear what drives microglial cells to the site of plaques in mice treated with the ApoBSecNEP; however, the increased accumulation of NEP with the plaques may be the signal for increased microglial activation. Previous studies have shown that monocyte derived macrophages are capable of taking up Aβ and degrading Aβ fibrils, however, fibrillar Aβ degradation in macrophages was sensitive to lysosomal and insulin degrading enzyme inhibitors but insensitive to proteasomal and neprilysin inhibitors[45]. Therefore, it is unlikely ApoBsecNEP detected in macrophages was directly involved in the degradation of Aβ fibrils but rather it might have increased the cleavage of neuropeptide substrates that promote leukocyte migration[46]. NEP has previously been shown to be expressed by leukocytes and process peptides involved in migration such bacterial chemotactic peptide N-formylmethionyl-leucyl-phenylalanine (fMLP). Inhibition of CD10/NEP on the surface of human neutrophils (PMNs) in vitro inhibits migration toward this chemotaxin, suggesting that enzymatic inactivation by NEP regulates the neutrophil response to fMLP[47].

The accumulation of ApoBSecNEP in the CNS of APPtg mice was significantly higher than in the non-tg controls. Since the levels of the targeted receptor on the BBB namely a member of the LDL receptor family was not different between control and tg mice then it is possible that other mechanisms might have been involved. One possibility is that the rate of accumulation of the recombinant protein in the CNS might depend on the Aβ burden in CNS. Increased trafficking of ApoBsecNEP into the CNS of APP tg mice might raise questions as to the potential for toxicity. In addition to peptides involved in chemoattraction, NEP has been shown to cleave neuroactive peptides such as met-enkephalin, substance P and neuropepetide Y. We have previously shown that regulated expression of NEP in the CNS is neuroprotective and does not result in a deleterious degradation of neuropeptides or trophic factors[30], [48]. Moreover, the present study showed that mice treated with the ApoBsecNEP displayed improved behavioral performance in water maze and amelioration of the synaptic pathology. Taken together these results suggest that targeted delivery of NEP across the BBB may be a viable therapy for AD. Although the studies here utilize the lentivirus vector for delivery of the gene for the recombinant protein to the mouse liver, it is likely that delivery of the ApoBSecNEP protein by intravenous injection would have similar effects. Thus, the ApoBSecNEP may be a feasible non-invasive therapeutic option for reducing the functional deficits and the accumulation of Aβ in AD.

## Materials and Methods

### Ethics Statement

All experiments described were carried out in strict accordance with good animal practice according to NIH recommendations, and all procedures for animal use were approved by the Institutional Animal Care and Use Committee at the University of California at San Diego (UCSD) under protocol #S07221.

### Lentivirus vector production

The pre-pro trypsin secretory signal was cloned to the 5′ of a vector expressing the human secreted neprilysin (aa 52–750). The vector was designated LV-SecNEP. The ApoB LDL-R binding domain (aa 3371- 3409)[21] was cloned with the secreted neprilysin between the secretory signal and the coding sequence for the human neprilysin producing LV-ApoBSecNEP (Figure S1A). As a control for transported proteins, a virus was generated as described above with eGFP, pre-pro trypsin secretory signal and the ApoB LDL-R binding domain (pLV-ApoBGFP). Lentiviruses expressing NEP, SecNEP, ApoBSecNEP, GFP or ApoBGFP were generated essentially as described[49]. An empty lentiviral vector (LV-control) was utilized for comparison purposes and as a control. Titers were determined by p24 ELISA assay (Perkin Elmer) as described[49].

### Neprilysin activity assays

The relative rate of Aβ processing by NEP was determined by using an N-terminal FITC-tagged human Aβ (Aβ_42_; rPeptide). For cell-free assays, 20 µg of cell lysates, 20 µl conditioned cell media or 100 ng of human recombinant NEP (R&D Systems) was incubated with 100 mM Tris, pH 7.4, 10 µM ZnCl_2_, and 33 µM FITC-labeled Aβ in a final volume of 50 µl. Thiorphan (1 mM; Calbiochem) was used as a NEP-specific protease inhibitor. Aliquots were taken at 0 and 24 hours, stopped with an equal volume of 8 M urea, run on 12% SDS-PAGE gels with MES buffer (Invitrogen), and analyzed with a Versadoc XL imaging apparatus (Bio-Rad). The proteolytic activity of NEP was measured as previously described[29] using the substrate 3-dansyl-D-Ala-Gly-p-(nitro)-Phe-Gly (DAGNPG; Sigma). Cell lysate was incubated with 50 µM DAGNPG and 1 µM captopril (to inhibit ACE cleavage of DAGNPG) in a volume of 200 µl at 37°C. Reactions were stopped by heating samples to 100°C for 5 min, then centrifuging. The supernatant was diluted into 50 mM Tris (pH 7.4) and fluorescence determined using a Victor2 multilabel plate reader (excitation 342 nm; emission 562 nm).

### Lentiviral infection of neuronal progenitor cells and challenge with Aβ

Adult rat hippocampal neural progenitor cells (NPCs) (Chemicon) were grown as previously described[50]. Briefly, NPCs were grown in DMEM/Ham's F-12 medium containing B27 supplements without Vitamin A (Gibco). Cells were plated onto glass coverslips and differentiated in DMEM/F12 medium containing N2 supplements (Gibco) for 4 days. The cells were then infected with LV-control, LV-NEP, LV-SecNEP or LV-ApoBSecNEP and after 24 hrs challenged with 10 nM Aβ_1–42_ (American Peptide) for 24 hours. One set of cells were grown in plates for analysis of NEP activity and protein levels in lysates and conditioned media and the other set were grown on coverslips and fixed in 4% PFA for immunohistochemistry. For this purpose, coverslips were immunolabeled with antibodies against NEP (Abcam), neuron specific tubulin-III (Millipore Corporation) and activated caspase-3 (Cell Signaling) followed by secondary antibodies tagged with tyramide red or FITC and imaged with the laser scanning confocal microscope. Images were analyzed with the NIH Image J program to assess levels of pixel intensity.

### Lentivirus injections into APP tg mice

The APP tg mice used in these studies express mutated human (h)APP751 under the control of the mThy-1 promoter (mThy1-hAPP751; line 41). These tg mice are unique in that, compared to other tg models, amyloid plaques are found in the cortex beginning at 3 months and in the hippocampus at 4 months of age[51]. In addition, the mThy1-hAPP751 mice show learning and memory deficit in the Morris water maze beginning at 6 months of age[28].

Following NIH guidelines for the humane treatment of animals, briefly, as described[21], APP tg mice or nontg control littermates (age 6 months) were injected intra-peritoneally with either the LV-control, LV-NEP, LV-SecNEP or LV-ApoBSecNEP (1×10^9^ transducing units (tdu)) in a total volume of 200 µl. For each group 8 mice were included for a total of 32 nontg and 32 APP tg mice. For control experiments of the recombinant hybrid protein trafficking into the CNS a subgroup of nontg (n = 4 each per group) and APP tg mice (n = 4 each per groups) were injected intra-peritoneally with LV-GFP or LV-ApoBGFP.

### Water maze testing

As previously described[28], in order to evaluate the functional effects of LV-ApoBSecNEP treatment in mice, groups of APP tg animals were tested in the water maze. For this purpose, a pool (diameter 180 cm) was filled with opaque water (24°C) and mice were first trained to locate a visible platform (days 1–3) and then a submerged hidden platform (days 4–7) in three daily trials 2–3 min apart. Mice that failed to find the hidden platform within 90 seconds were placed on it for 30 seconds. The same platform location was used for all sessions and all mice. The starting point at which each mouse was placed into the water was changed randomly between two alternative entry points located at a similar distance from the platform. On day 8, another visible platform trial was performed to exclude differences in motivation and fatigue. Time to reach the platform (latency), path length, and swim speed were recorded with a Noldus Instruments EthoVision video tracking system (San Diego Instruments) set to analyze two samples per second.

### Animal maintenance and tissue processing

Four weeks after the lentiviral intra-peritoneal injections, mice were tested in the water maze and then anesthetized, perfused with cold saline, and their brains removed. The left hemibrain was frozen in isopentane cooled in a Histobath (Shandon Lipshaw, Pittsburgh, PA) and was used for western blot analysis. The right hemibrain was immersion-fixed in 4% PFA in PBS, pH 7.4. Fixed hemibrains were serially sectioned at 40 µm with a vibratome (Leica) for immunocytochemical and LSCM analysis.

### Tissue and cell fractionation and western blot analysis

Samples were homogenized and separated into cytosolic (soluble) and membrane (insoluble) fractions as described[23], [38]. Briefly, mouse brain samples (0.1 g) was homogenized in 0.4 ml of solution containing containing PBS [pH 7.4], 0.32 M sucrose, 50 mM HEPES, 25 mM MgCl_2_, 0.5 mM DTT, 200 µg/ml PMSF, 2 µg/ml pepstatin A, 4 µg/ml leupeptin, 30 µg/ml benzamidine hydrochloride (Calbiochem, San Diego, CA). The samples were centrifuged at 1,000× g for 10 minutes at 4°C. Supernatants were retained and placed into appropriate ultra-centrifuge tubes and the pellets were re-homogenized in 0.3 ml of Buffer A and re-centrifuged at 1,000× g for 10 minutes at 4°C. The second supernatant was collected and combined with the first supernatant and centrifuged at 100,000× g for one hour at 4°C. This final supernatant was collected to serve as the cytosolic fraction and the remaining pellet was resuspended in 0.2 ml of buffer and re-homogenized; this was the membrane fraction. Protein concentrations were quantified by BCA (Pierce). Levels of APP and Aβ immunoreactivity were determined in brain homogenates by western blot as described previously[38], [52] utilizing the membrane fractions. Antibodies against full-length (FL) APP (mouse monoclonal, clone 22C11, Chemicon), Aβ - 82E1 (IBL International) and NEP (mouse monoclonal, clone 56C6, Abcam) followed by secondary antibodies tagged with horseradish peroxidase (HRP) (Santa Cruz Biotechnology) were used and then visualized by enhanced chemiluminescence and analyzed with a Versadoc XL imaging apparatus (Bio-Rad). Analyses of actin levels were used as a loading control.

### Analysis of Aβ and plaque load by immunocytochemistry

To evaluate the amyloid plaque load, briefly as previously described[28], vibratome sections were incubated overnight at 4°C with the mouse monoclonal antibody against Aβ (clone 82E1, IBL international) followed by fluorescein isothiocyanate (FITC)-conjugated anti-mouse IgG (Vector Laboratories). The FITC-labeled sections were imaged with the laser scanning confocal microscope (LSCM, MRC1024, BioRad) as described previously[27]. Digitized images were analyzed with the NIH Image 1.43 program to determine the percent area of the neuropil occupied by Aβ-immunoreactive deposits in the frontal cortex and hippocampus. To evaluate effects on intracellular Aβ, sections were immunolabeled with the mouse monoclonal antibody against Aβ (clone 4G8; Senetek) and with the antibody against the N-terminus of Aβ (aa 1–16; clone 82E1; Immunobiological Llaboratorie Co.) followed by incubation with secondary biotinylated anti-mouse IgG and then ABC and DAB. Sections were transferred to SuperFrost slides (Fisher Scientific) and mounted under glass coverslips with anti-fading media (Vector Laboratories). All sections were processed under the same standardized conditions. Three immunolabeled sections were analyzed per mouse and the average of individual measurements was used to calculate group means.

### Analysis of NEP expression, double immunolabeling and neurodegeneration

To verify the expression levels of NEP, vibratome sections were immunolabeled with a monoclonal antibody against NEP (CD10, Abcam) and detected with the Tyramide Signal Amplification™-Direct (Red) system (NEN Life Sciences,). To evaluate the co-localization of NEP, double immunocytochemical analysis was performed as previously described[53]. For this purpose, vibratome sections were immunolabeled with a monoclonal antibody against NEP (Abcam) detected with the Tyramide Signal Amplification™-Direct (Red) system (NEN Life Sciences) and the mouse monoclonal antibodies against NeuN (Millipore), MAP2 (Millipore) and Aβ (clone 82E1) detected with FITC-conjugated secondary antibodies (Vector Laboratories)[53]. Additional co-localization studies were performed with antibodies against Aβ (clone 82E1) and microglia/macrophage markers Iba-1 (Wako), CD68 (Abcam) and CD11b (Abcam). All sections were processed simultaneously under the same conditions, and experiments were performed twice to assess reproducibility. Sections were imaged with a Zeiss 63X (N.A. 1.4) objective on an Axiovert 35 microscope (Zeiss) with an attached MRC1024 LSCM system (BioRad)[53]. To confirm the specificity of primary antibodies, control experiments were performed where sections were incubated overnight in the absence of primary antibody (deleted) or preimmune serum and primary antibody alone.

The integrity of the neuronal structure was evaluated as previously described[28], [54]; briefly, blind-coded, 40 µm thick vibratome sections from mouse brains fixed in 4% paraformaldehyde were immunolabeled with the mouse monoclonal antibodies against SNAP25 (synaptic marker, Millipore) and PSD95 (postsynaptic marker, Abcam). After overnight incubation, sections were incubated with the Tyramide Signal Amplification™-Direct (Red) system (NEN Life Sciences) transferred to SuperFrost slides (Fisher Scientific) and mounted under glass coverslips with anti-fading media (Vector Laboratories). All sections were processed under the same standardized conditions. The immunolabeled blind-coded sections were serially imaged with the LSCM (MRC1024, BioRad) and analyzed with the Image 1.43 program (NIH), as previously described[55]. For each mouse, a total of 3 sections were analyzed and for each section, 4 fields in the frontal cortex and hippocampus were examined. Results were expressed as percent area of the neuropil occupied.

### Statistical analyses

Analyses were carried out with the StatView 5.0 program (SAS Institute Inc., Cary, NC). Differences among means were assessed by one-way ANOVA with post-hoc Dunnett's or Tukey-Kramer tests. All values in the figures are expressed as means ±SEM. Comparisons between 2 groups were done with the unpaired two-tailed Student's t-test. Correlation studies were carried out by simple regression analysis and the null hypothesis was rejected at the 0.05 level.

## Supporting Information

Figure S1 Characterization of lentiviral vectors containing the neprilysin constructs. (A) Diagrammatic representation of the secreted ApoBSecNEP cDNA inserted into the 3^rd^ generation lentivirus vector. (B) Immunoblot analysis for expression levels of NEP, GFP and actin from the LV-control, LV-NEP, LV-SecNEP, LV-ApoBSecNEP and LV-ApoBGFP vectors. The 293T cells were infected and 24 hrs later analysis were performed with cell lysates (20 µg, CL) and conditioned media (20 µl, CM). (C–G) B103 neuronal cells infected with the lentivirus vectors and immunostained for expression of NEP (C–F) or visualized for GFP expression (G). Scale bars  = 25 µm.
(TIF)

Figure S2 Patterns of ApoBGFP distribution in the CNS following peripheral delivery. Lentiviral vectors were delivered by intra-peritoneal injection to non-tg mice and 4 weeks later brains were visualized for the accumulation of GFP protein. (A, B) Low power view of the GFP distribution in the neocortex and hippocampus for LV-control or LV-ApoBGFP vectors respectively (Scale bar  = 200 µm for A,B). (C, D) High power demonstrating the accumulation of the ApoBGFP in the dentate granular cells of the hippocampus (Scale bar  = 20 µm for C, D). n = 4 mice per group.
(TIF)

Figure S3 Immunoblot analysis of the levels of neprilysin accumulation in the CNS following intra-peritoneal delivery of LV-ApoBSecNEP. The posterior cortex and hippocampus from each mouse were dissected and homogenized. (A) Representative immunoblot with total protein from the CNS following intra-peritoneal delivery of LV-control, LV-NEP, LV-SecNEP or LV-ApoBSecNEP respectively analyzed with antibodies against NEP and actin. (B) Computer aided image analysis of the NEP immunoreactive band for nontg and APP tg mice that received the lentiviral vectors (B). *  =  indicates statistically significant difference by 1-way ANOVA with poshoc Dunnet's when compared to nontg treated animals (p<0.05). n = 8 mice per group.
(TIF)

Figure S4 Co-localization of Aβ with macrophage/microglial cell markers in APP tg mice treated with LV-ApoBSecNEP. For these studies vibratome sections from APP tg mice were double labeled with antibodies against Aβ (green), or the microglial markers, Iba1 (red) or CD11b (red) and analyzed with the laser scanning confocal microscope. DAPI (blue) was used to visualize nuclei. Images are from plaques distributed in the hippocampus. (A–F) Double labeling analysis with antibodies against Aβ and Iba1 in mice treated with LV-control or LV-ApoBSecNEP respectively. (G–L) Double labeling analysis with antibodies against Aβ and CD11b in mice treated with LV-control or LV-ApoBSecNEP respectively. Arrows indicate areas of colocalization. Scale bar  = 50 µm.
(TIF)
