# Correction: Peripheral Delivery of a CNS Targeted, Metalo-Protease Reduces Aβ Toxicity in a Mouse Model of Alzheimer’s Disease

**DOI:** 10.1371/journal.pone.0330647

**Published:** 2025-08-20

**Authors:** Brian Spencer, Robert A. Marr, Ryan Gindi, Rewati Potkar, Sarah Michael, Anthony Adame, Edward Rockenstein, Inder M. Verma, Eliezer Masliah

After this article [1] was published, concerns were raised that data shown in Fig 5I, 5K of this article [1] appear morphologically similar to data shown in Fig 3I, 3J, respectively, of [2]. The authors clarify that incorrect panels were used during the preparation of Fig 5I and 5K, and they provided an updated Fig 5 with the correct images from the original experiment. The prior article [2] was published by the Society for Neuroscience and is not offered under a CC BY license, therefore the original Fig 5I and 5K are removed from this article’s CC BY license.

**Fig 5:**
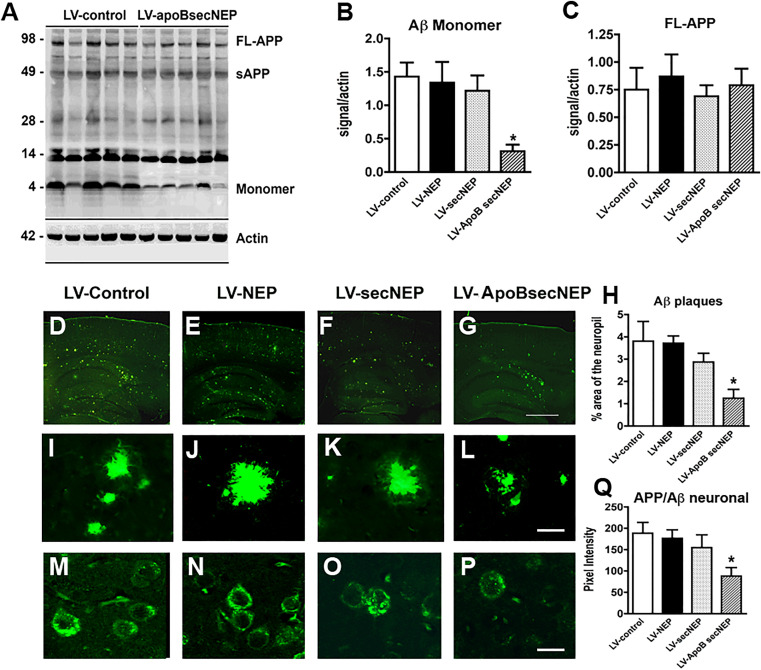
ApoBSecNEP reduces Aβ in the CNS of APP tg mice. Sections from APP tg mice that had received peripheral injections with the lentiviral vectors were homogenized, fractioned and examined for levels of APP and Aβ by western blot. **(A)** Representative immunoblot probed with the 82E1 monoclonal antibody displaying the reduction in the levels of Aβ in APP tg mice treated with LV-ApoBSecNEP. **(B–C)** Analysis of immunoblot for levels of Aβ monomers and full length APP. **(D–G)** Low magnification view of sections from APP tg mice treated with LV-control, LV-NEP, LV-SecNEP or LV-ApoBSecNEP respectively and immunolabeled with a monoclonal antibody against Aβ (82E1) imaged with the laser scanning microscope. **(H)** Computer aided image analysis of the % area of the neuropil occupied by Aβ immunoreactive deposits. **(I–L)** Higher magnification view of the Aβ immunoreactive plaques in APP tg mice treated with the various lentiviruses. **(M–P)** Representative images of the patterns of intraneuronal APP/Aβ immunostaining in the frontal cortex from APP tg mice treated with LV-Control, LV-NEP, LV-SecNEP or LV-ApoBSecNEP respectively immunolabeled with a monoclonal antibody against Aβ (82E1) imaged with the laser scanning microscope. **(Q)** Image analysis of levels of intracellular APP/Aβ immunostaining. Scale bar = 15 µm for I-L and 10 µm for M-P. * - indicates statistically significant difference by 1-way ANOVA with poshoc Dunnet’s when compared to LV-Control treated animals (*p* < 0.05). n = 8 mice per group.

In reviewing this matter, PLOS also noted that there does not appear to be blot image data in the lower half of the Fig 5A Actin panel, and in the Fig S1B Actin blot the background appears uniform and there are abrupt discontinuities around some of the bands. The *PLOS One* Editors concluded that the Fig 5A issue does not impact the reliability of the published findings. PLOS was unable to verify the integrity of the Fig S1B Actin blot due to the poor resolution of the published figure and unavailability of original image data, and so readers are advised to interpret Fig S1B results with caution.

The available underlying data for Figs 2, 5, 6, S1, and S4 are provided in the S1–S8 Files. The uncropped image data underlying the western blot results in Figs 5 and S1 are no longer available.

## Supporting information

S1 File Individual level data underlying Fig 1C and 1D. (XLSX)

S2 File Image data underlying Fig 2A. The image underlying the Fig 2 Tubulin LV-control Aβ CM (10 nM) result appears blurred in the lower right area where the scale bar was placed for the published figure. There also appear to be black markings near the lower and left edges of the image, which are also visible in the published figure if levels are adjusted to visualize background.
(ZIP)

S3 File Individual level data underlying Fig 2B–2E. (XLSX)

S4 File Individual level data underlying Fig 5B and 5C. (XLSX)

S5 File Image data underlying Fig 5I–5P. (ZIP)

S6 File Individual level data underlying Fig 5H and 5Q. (XLSX)

S7 File Image data underlying Fig 6C, 6F, and 6G. (ZIP)

S8 File Image data underlying Fig S4C, S4F, S4I, and S4L. (ZIP)
