# Supplementary figures and images for: Peripheral Delivery of a CNS Targeted, Metalo-Protease Reduces Aβ Toxicity in a Mouse Model of Alzheimer's Disease
Source: PLoS One. 2011 Jan 31;6(1):e16575. doi: 10.1371/journal.pone.0016575 (PMC3031588; doi:10.1371/journal.pone.0016575)

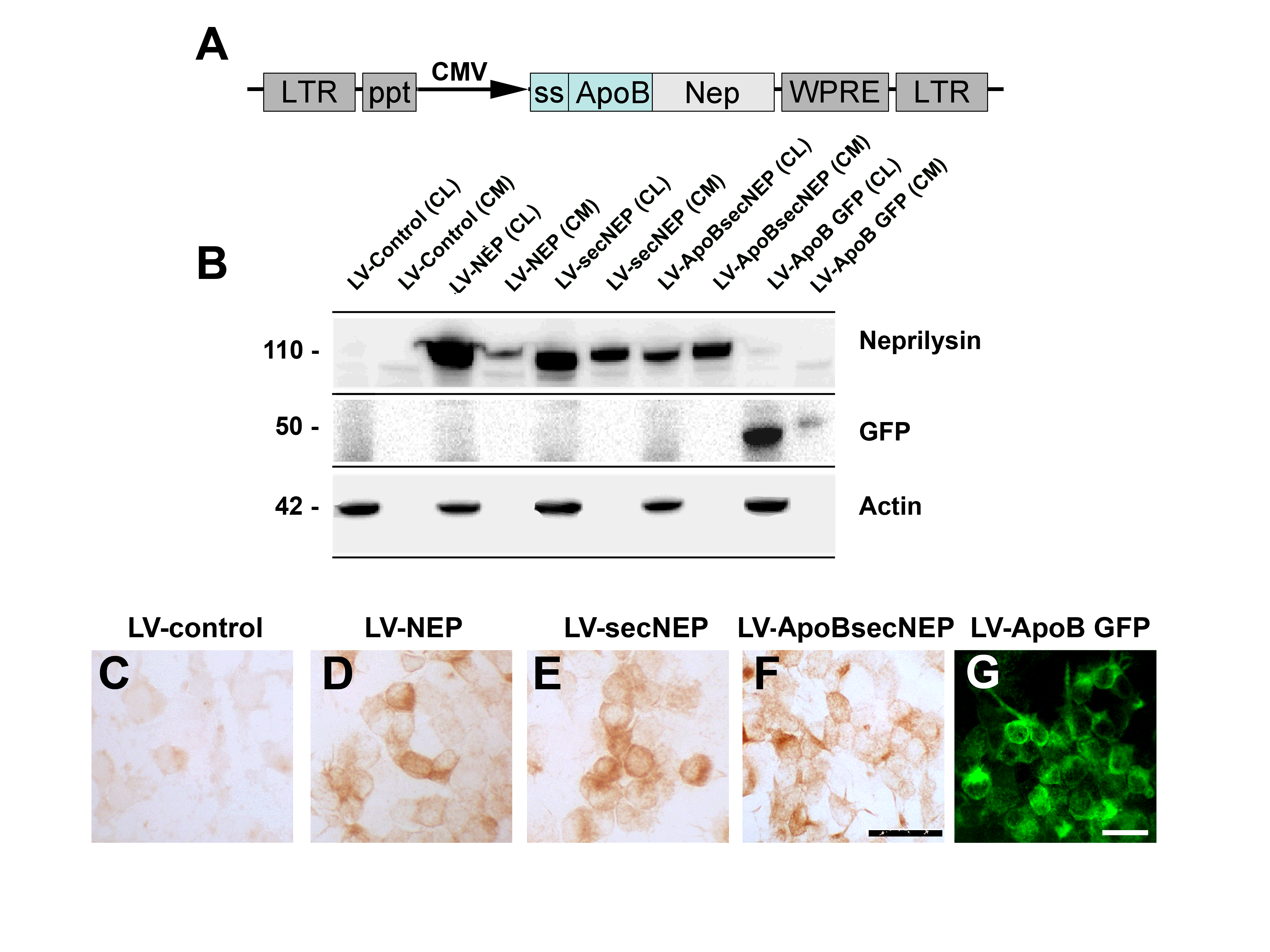

Supplement: Figure S1 — Characterization of lentiviral vectors containing the neprilysin constructs. (A) Diagrammatic representation of the secreted ApoBSecNEP cDNA inserted into the 3rd generation lentivirus vector. (B) Immunoblot analysis for expression levels of NEP, GFP and actin from the LV-control, LV-NEP, LV-SecNEP, LV-ApoBSecNEP and LV-ApoBGFP vectors. The 293T cells were infected and 24 hrs later analysis were performed with cell lysates (20 µg, CL) and conditioned media (20 µl, CM). (C–G) B103 neuronal cells infected with the lentivirus vectors and immunostained for expression of NEP (C–F) or visualized for GFP expression (G). Scale bars = 25 µm. (TIF) [file pone.0016575.s001.tif]

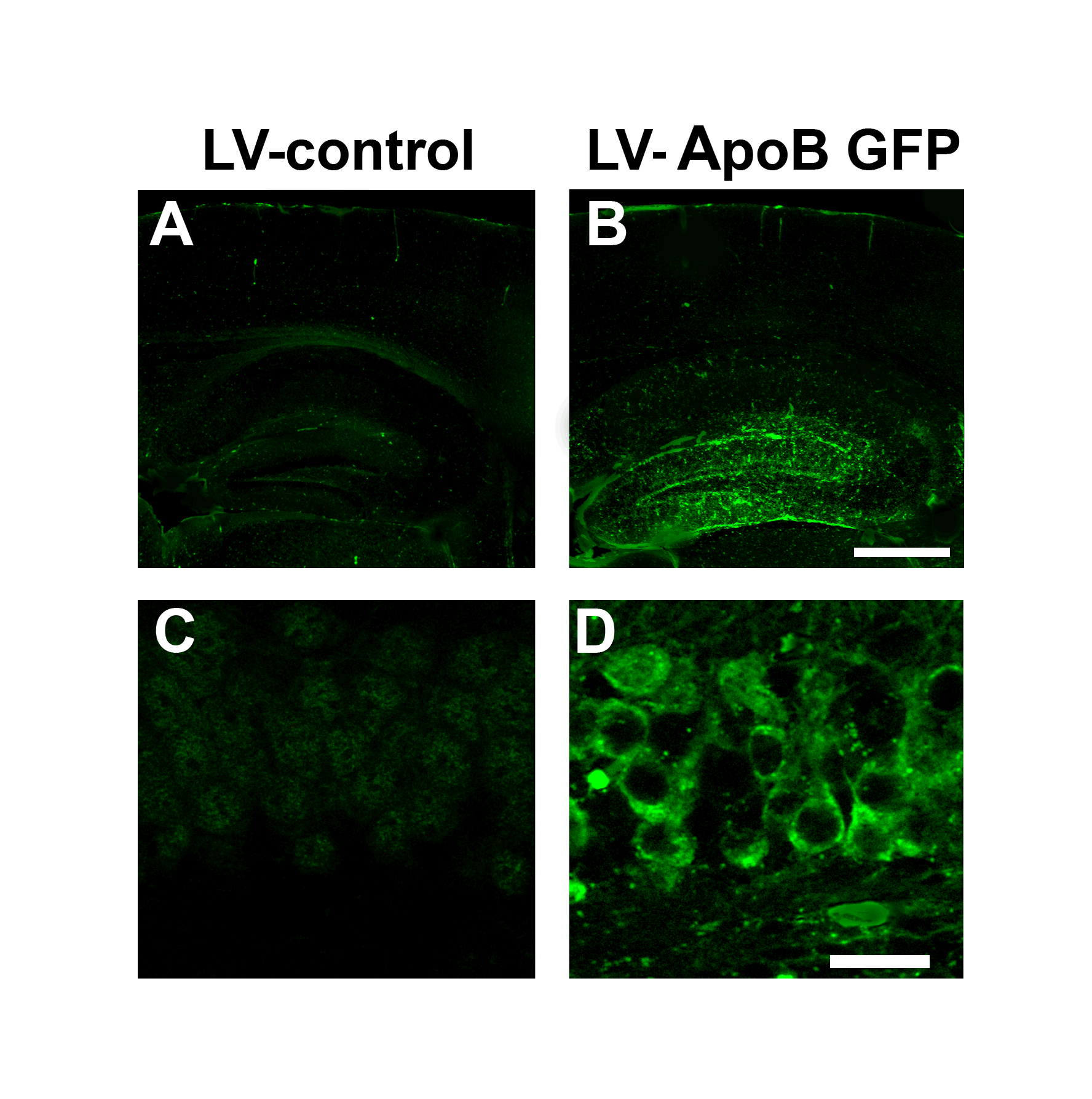

Supplement: Figure S2 — Patterns of ApoBGFP distribution in the CNS following peripheral delivery. Lentiviral vectors were delivered by intra-peritoneal injection to non-tg mice and 4 weeks later brains were visualized for the accumulation of GFP protein. (A, B) Low power view of the GFP distribution in the neocortex and hippocampus for LV-control or LV-ApoBGFP vectors respectively (Scale bar = 200 µm for A,B). (C, D) High power demonstrating the accumulation of the ApoBGFP in the dentate granular cells of the hippocampus (Scale bar = 20 µm for C, D). n = 4 mice per group. (TIF) [file pone.0016575.s002.tif]

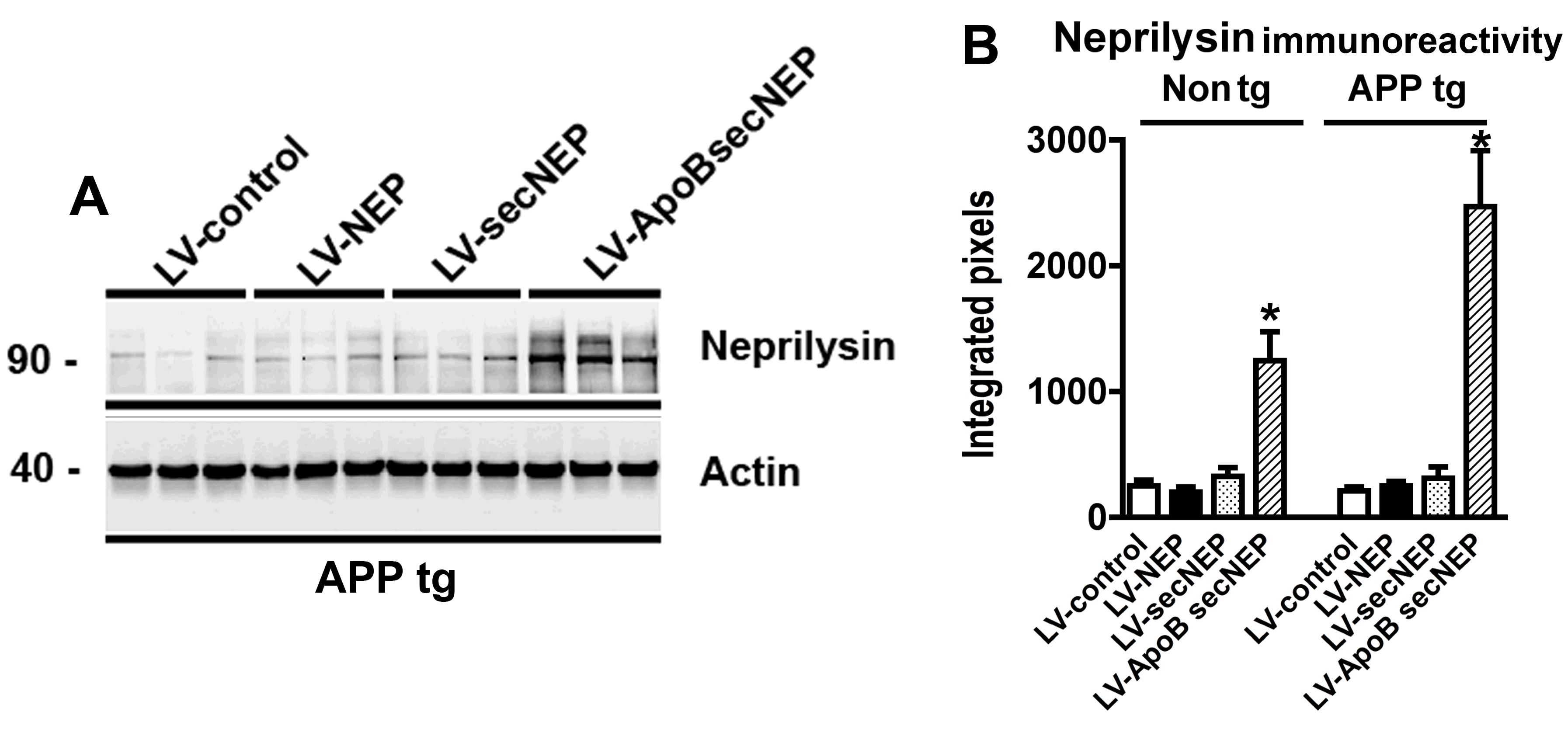

Supplement: Figure S3 — Immunoblot analysis of the levels of neprilysin accumulation in the CNS following intra-peritoneal delivery of LV-ApoBSecNEP. The posterior cortex and hippocampus from each mouse were dissected and homogenized. (A) Representative immunoblot with total protein from the CNS following intra-peritoneal delivery of LV-control, LV-NEP, LV-SecNEP or LV-ApoBSecNEP respectively analyzed with antibodies against NEP and actin. (B) Computer aided image analysis of the NEP immunoreactive band for nontg and APP tg mice that received the lentiviral vectors (B). * = indicates statistically significant difference by 1-way ANOVA with poshoc Dunnet's when compared to nontg treated animals (p<0.05). n = 8 mice per group. (TIF) [file pone.0016575.s003.tif]

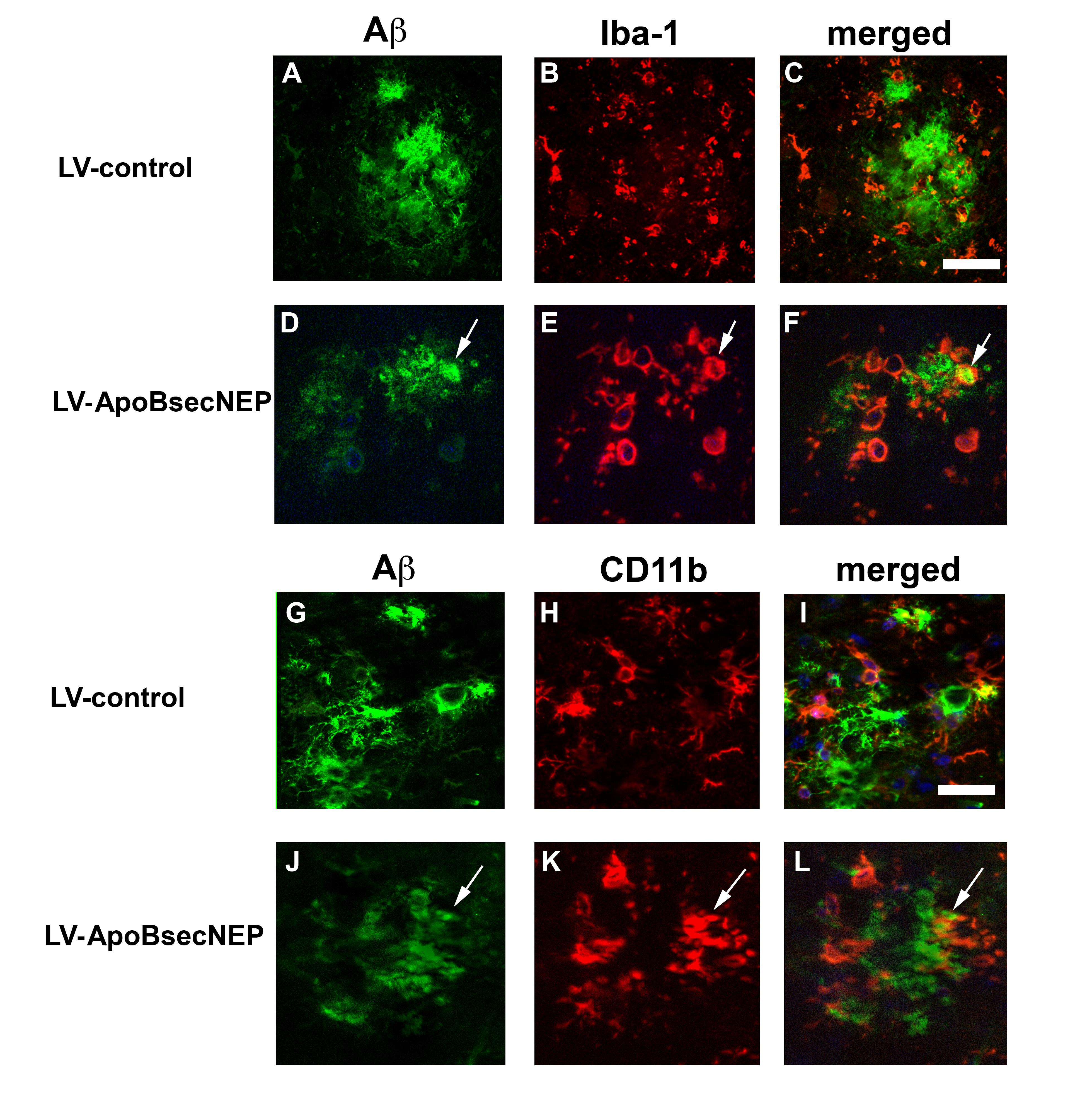

Supplement: Figure S4 — Co-localization of Aβ with macrophage/microglial cell markers in APP tg mice treated with LV-ApoBSecNEP. For these studies vibratome sections from APP tg mice were double labeled with antibodies against Aβ (green), or the microglial markers, Iba1 (red) or CD11b (red) and analyzed with the laser scanning confocal microscope. DAPI (blue) was used to visualize nuclei. Images are from plaques distributed in the hippocampus. (A–F) Double labeling analysis with antibodies against Aβ and Iba1 in mice treated with LV-control or LV-ApoBSecNEP respectively. (G–L) Double labeling analysis with antibodies against Aβ and CD11b in mice treated with LV-control or LV-ApoBSecNEP respectively. Arrows indicate areas of colocalization. Scale bar = 50 µm. (TIF) [file pone.0016575.s004.tif]
