# Supplementary figures and images for: Correction: Peripheral Delivery of a CNS Targeted, Metalo-Protease Reduces Aβ Toxicity in a Mouse Model of Alzheimer’s Disease
Source: PLoS One. 2025 Aug 20;20(8):e0330647. doi: 10.1371/journal.pone.0330647 (PMC12367130; doi:10.1371/journal.pone.0330647)

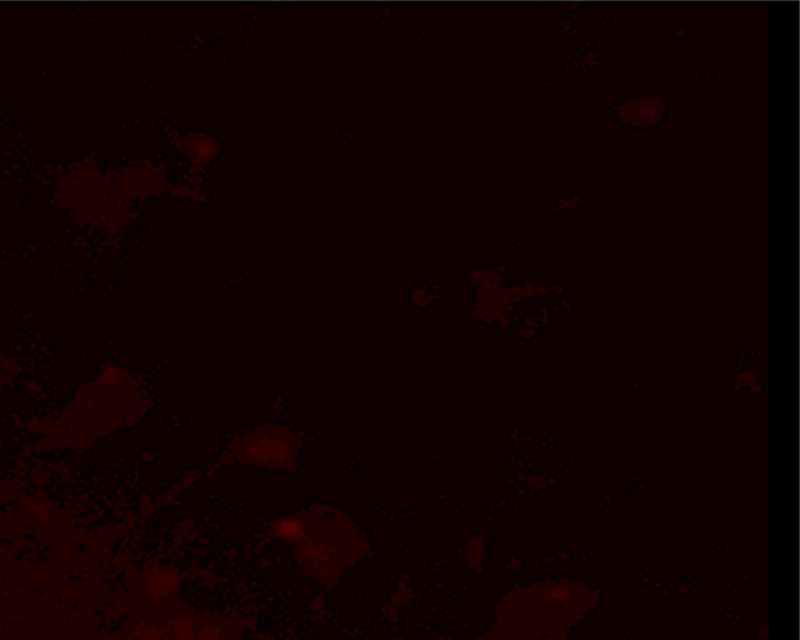

Supplement: S2 File — The image underlying the Fig 2 Tubulin LV-control Aβ CM (10 nM) result appears blurred in the lower right area where the scale bar was placed for the published figure. There also appear to be black markings near the lower and left edges of the image, which are also visible in the published figure if levels are adjusted to visualize background. (ZIP) [file pone.0330647.s002.zip › S2 File - underlying data Fig 2A/apobsecnep cells nep LV-control.tif]

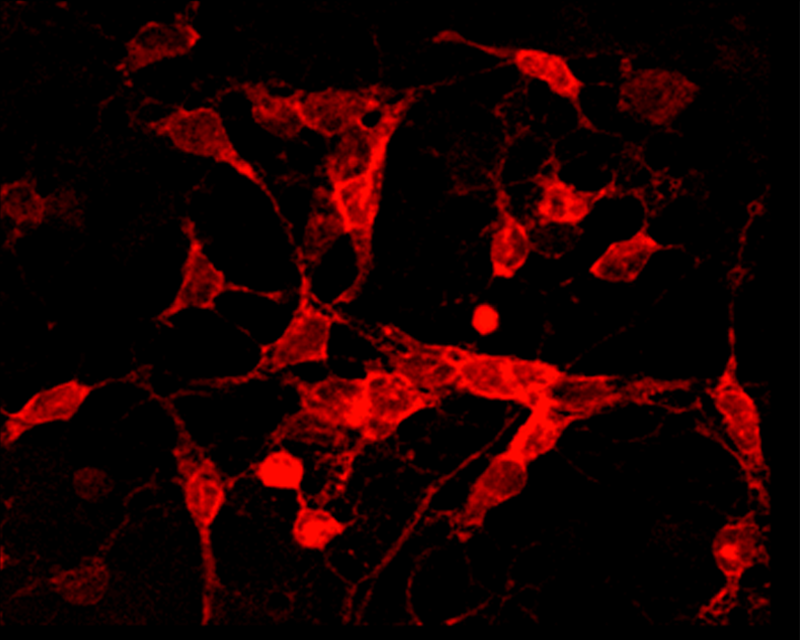

Supplement: S2 File — The image underlying the Fig 2 Tubulin LV-control Aβ CM (10 nM) result appears blurred in the lower right area where the scale bar was placed for the published figure. There also appear to be black markings near the lower and left edges of the image, which are also visible in the published figure if levels are adjusted to visualize background. (ZIP) [file pone.0330647.s002.zip › S2 File - underlying data Fig 2A/apobsecnep cells nep LV-Nep.tif]

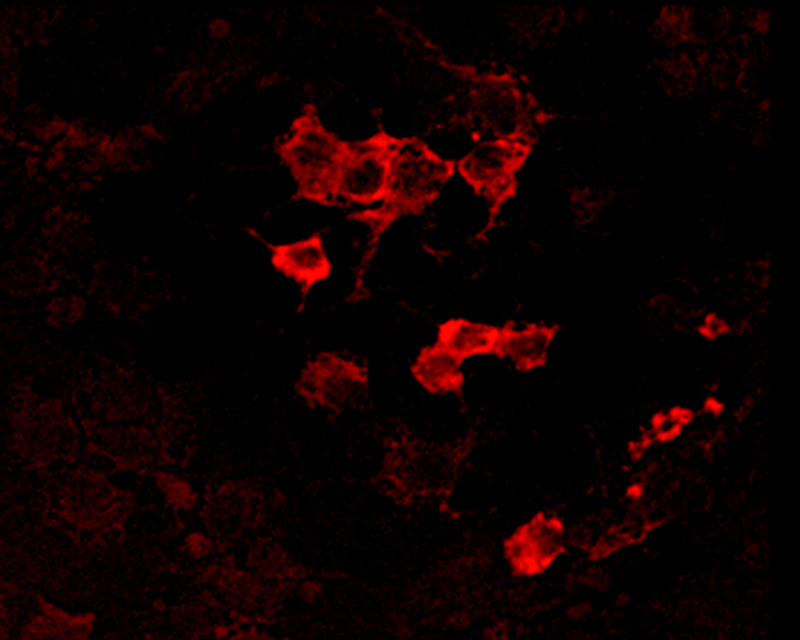

Supplement: S2 File — The image underlying the Fig 2 Tubulin LV-control Aβ CM (10 nM) result appears blurred in the lower right area where the scale bar was placed for the published figure. There also appear to be black markings near the lower and left edges of the image, which are also visible in the published figure if levels are adjusted to visualize background. (ZIP) [file pone.0330647.s002.zip › S2 File - underlying data Fig 2A/apobsecnep cells nep LV-secnep.tif]

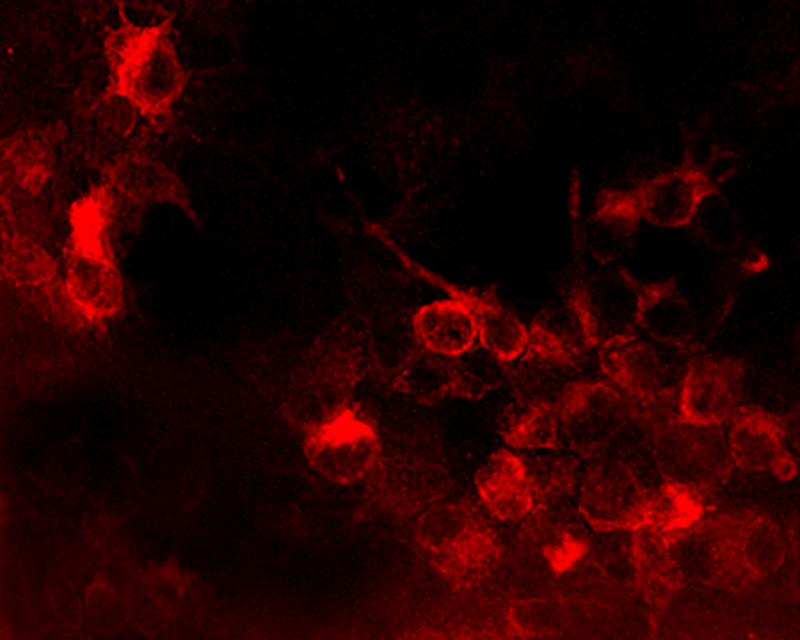

Supplement: S2 File — The image underlying the Fig 2 Tubulin LV-control Aβ CM (10 nM) result appears blurred in the lower right area where the scale bar was placed for the published figure. There also appear to be black markings near the lower and left edges of the image, which are also visible in the published figure if levels are adjusted to visualize background. (ZIP) [file pone.0330647.s002.zip › S2 File - underlying data Fig 2A/apobsecnep cells nep LV-secnepapob.tif]

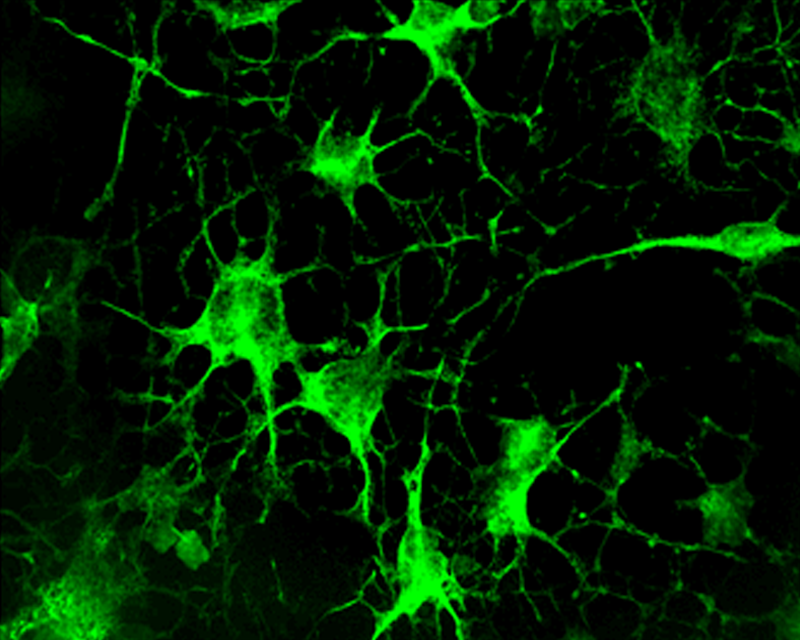

Supplement: S2 File — The image underlying the Fig 2 Tubulin LV-control Aβ CM (10 nM) result appears blurred in the lower right area where the scale bar was placed for the published figure. There also appear to be black markings near the lower and left edges of the image, which are also visible in the published figure if levels are adjusted to visualize background. (ZIP) [file pone.0330647.s002.zip › S2 File - underlying data Fig 2A/apobsecnep cells tub lv-apobsecnep Abeta.tif]

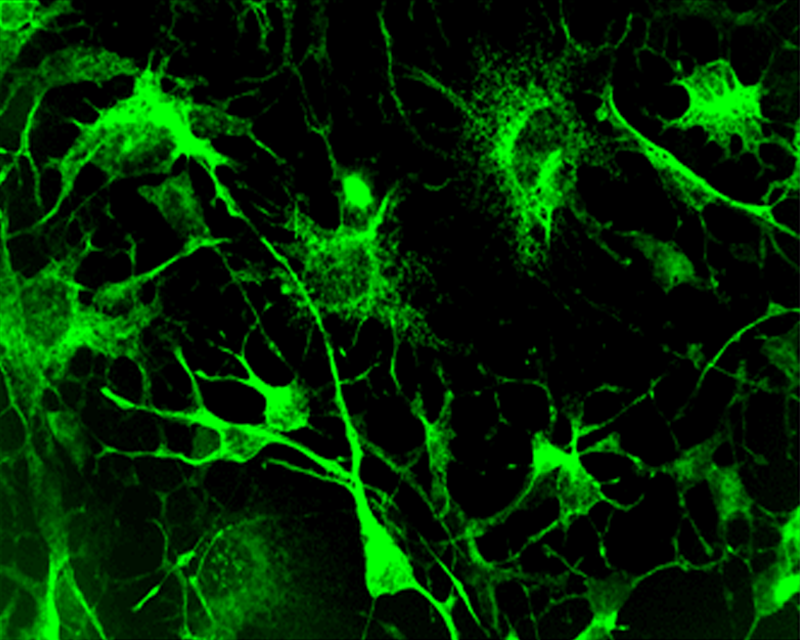

Supplement: S2 File — The image underlying the Fig 2 Tubulin LV-control Aβ CM (10 nM) result appears blurred in the lower right area where the scale bar was placed for the published figure. There also appear to be black markings near the lower and left edges of the image, which are also visible in the published figure if levels are adjusted to visualize background. (ZIP) [file pone.0330647.s002.zip › S2 File - underlying data Fig 2A/apobsecnep cells tub lv-apobsecnep veh.tif]

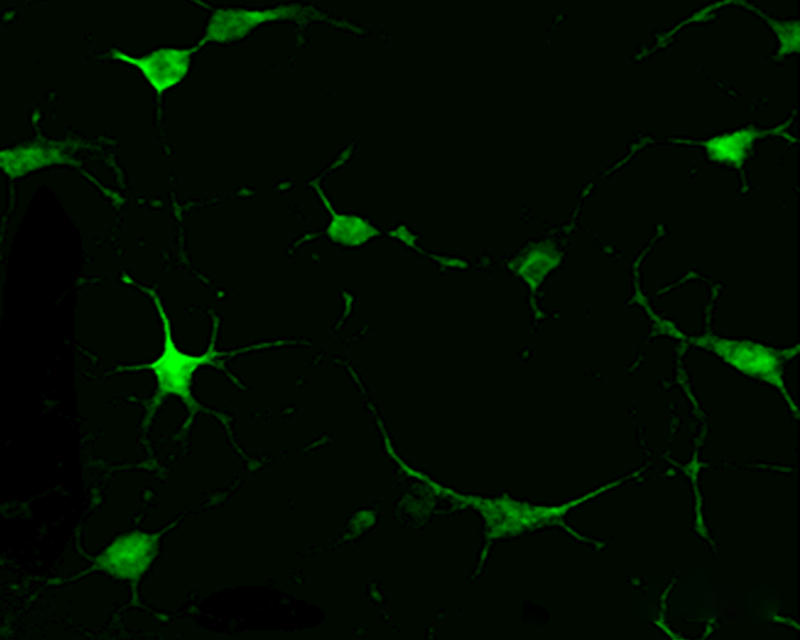

Supplement: S2 File — The image underlying the Fig 2 Tubulin LV-control Aβ CM (10 nM) result appears blurred in the lower right area where the scale bar was placed for the published figure. There also appear to be black markings near the lower and left edges of the image, which are also visible in the published figure if levels are adjusted to visualize background. (ZIP) [file pone.0330647.s002.zip › S2 File - underlying data Fig 2A/apobsecnep cells tub lv-ctl Abeta.tif]

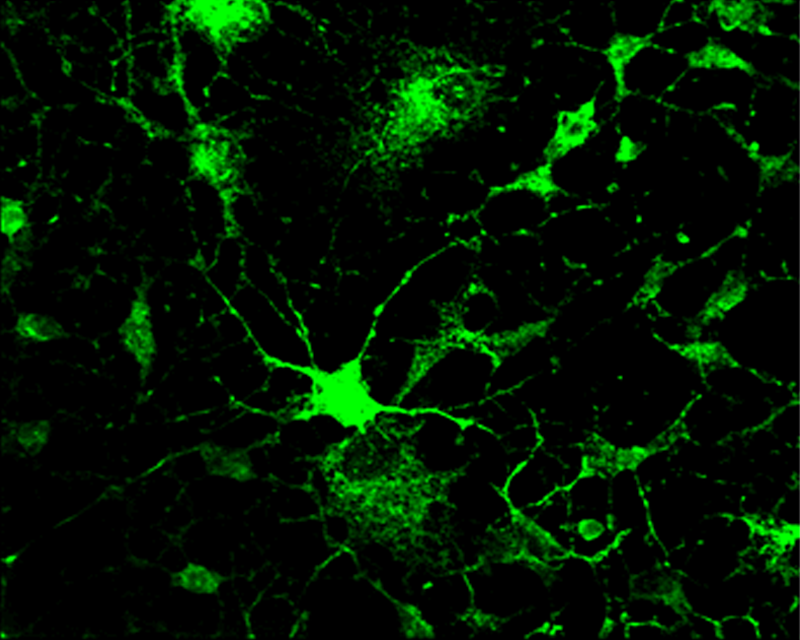

Supplement: S2 File — The image underlying the Fig 2 Tubulin LV-control Aβ CM (10 nM) result appears blurred in the lower right area where the scale bar was placed for the published figure. There also appear to be black markings near the lower and left edges of the image, which are also visible in the published figure if levels are adjusted to visualize background. (ZIP) [file pone.0330647.s002.zip › S2 File - underlying data Fig 2A/apobsecnep cells tub lv-ctl veh.tif]

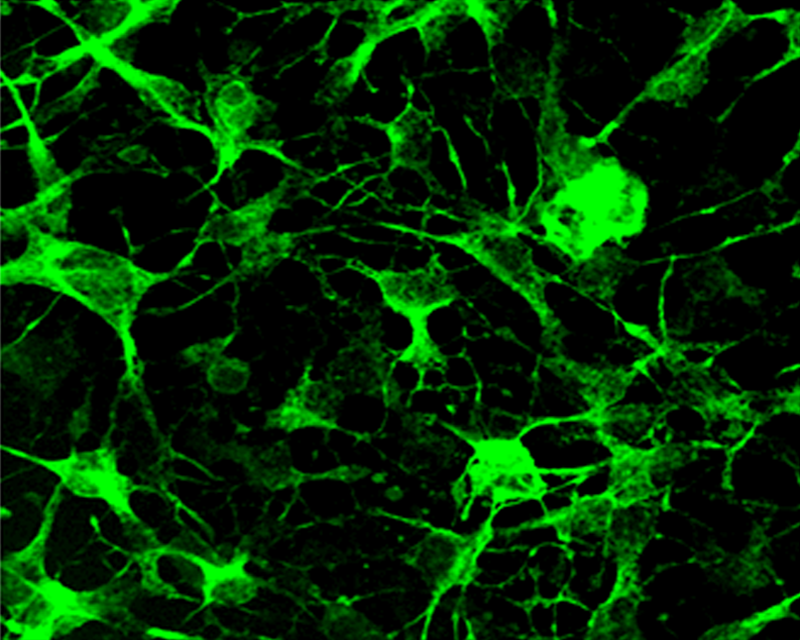

Supplement: S2 File — The image underlying the Fig 2 Tubulin LV-control Aβ CM (10 nM) result appears blurred in the lower right area where the scale bar was placed for the published figure. There also appear to be black markings near the lower and left edges of the image, which are also visible in the published figure if levels are adjusted to visualize background. (ZIP) [file pone.0330647.s002.zip › S2 File - underlying data Fig 2A/apobsecnep cells tub lv-nep Abeta.tif]

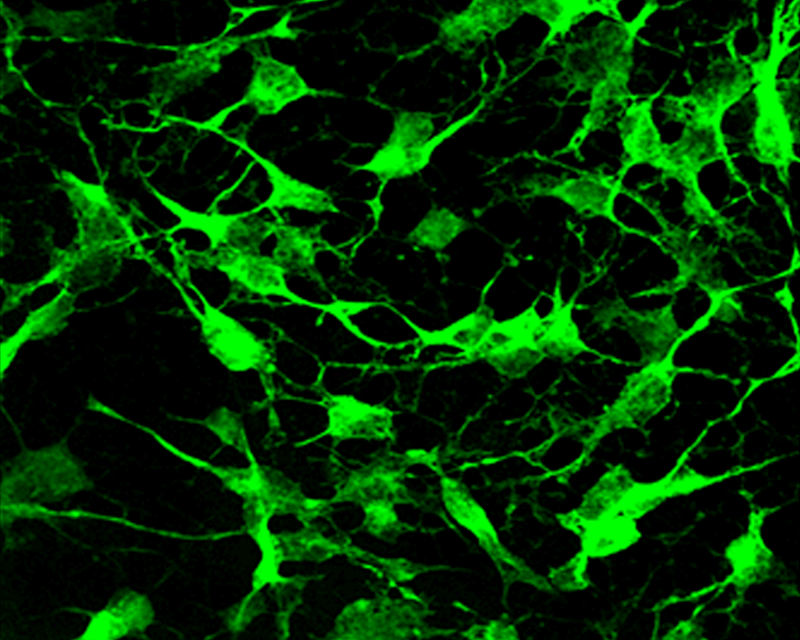

Supplement: S2 File — The image underlying the Fig 2 Tubulin LV-control Aβ CM (10 nM) result appears blurred in the lower right area where the scale bar was placed for the published figure. There also appear to be black markings near the lower and left edges of the image, which are also visible in the published figure if levels are adjusted to visualize background. (ZIP) [file pone.0330647.s002.zip › S2 File - underlying data Fig 2A/apobsecnep cells tub lv-nep veh.tif]

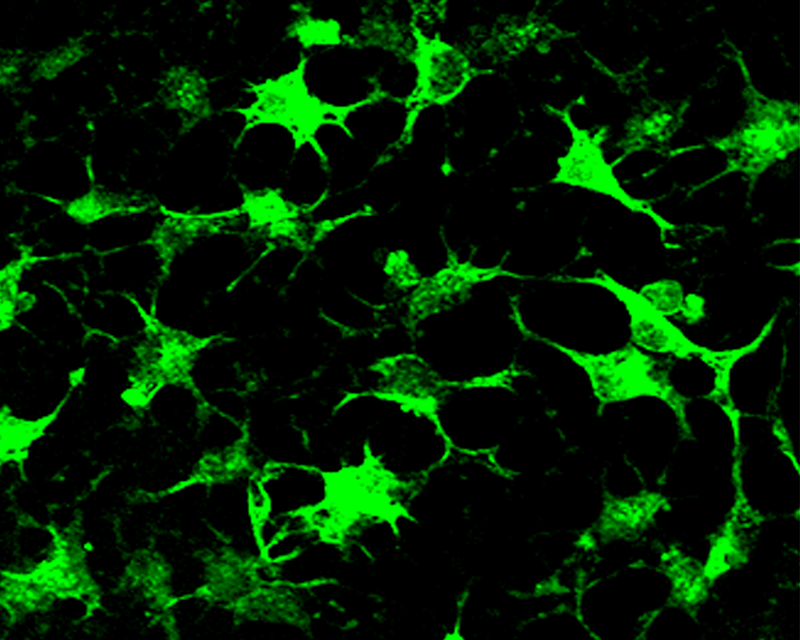

Supplement: S2 File — The image underlying the Fig 2 Tubulin LV-control Aβ CM (10 nM) result appears blurred in the lower right area where the scale bar was placed for the published figure. There also appear to be black markings near the lower and left edges of the image, which are also visible in the published figure if levels are adjusted to visualize background. (ZIP) [file pone.0330647.s002.zip › S2 File - underlying data Fig 2A/apobsecnep cells tub lv-secnep Abeta.tif]

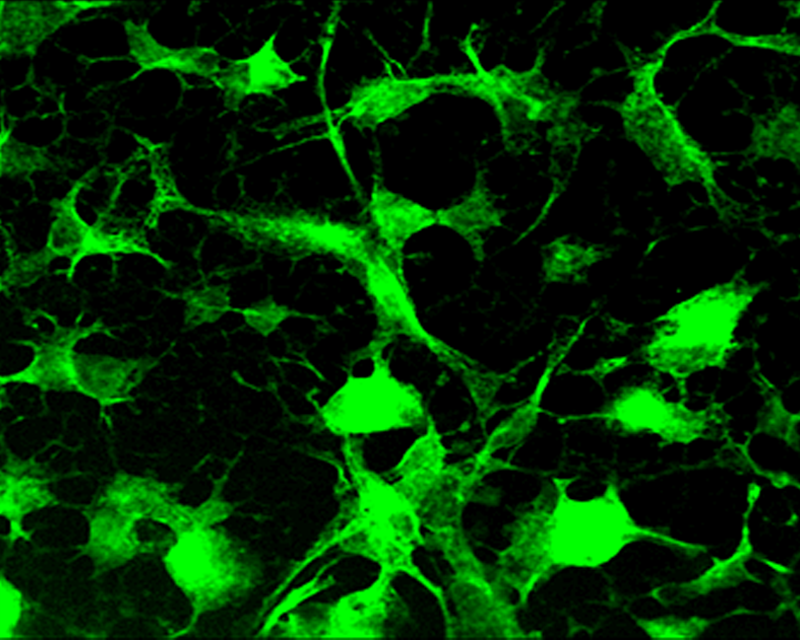

Supplement: S2 File — The image underlying the Fig 2 Tubulin LV-control Aβ CM (10 nM) result appears blurred in the lower right area where the scale bar was placed for the published figure. There also appear to be black markings near the lower and left edges of the image, which are also visible in the published figure if levels are adjusted to visualize background. (ZIP) [file pone.0330647.s002.zip › S2 File - underlying data Fig 2A/apobsecnep cells tub lv-secnep veh.tif]

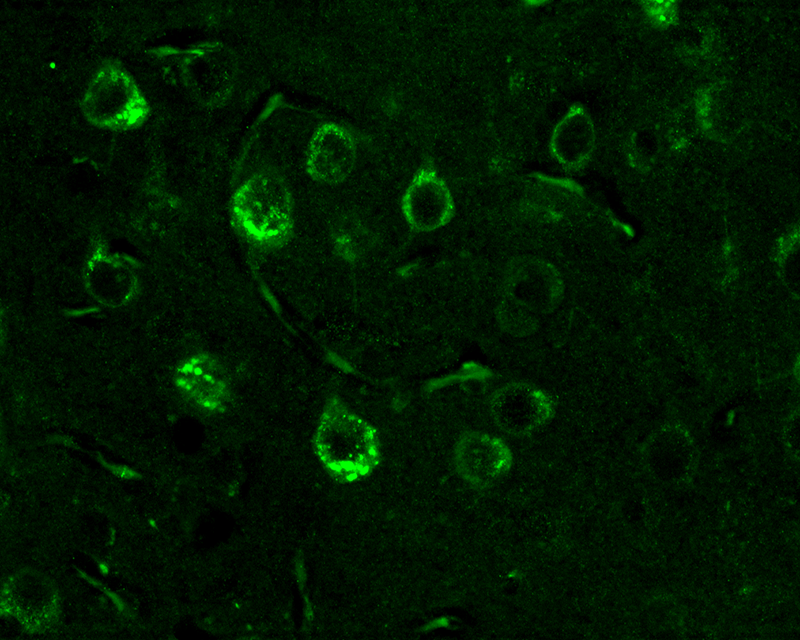

Supplement: S5 File — (ZIP) [file pone.0330647.s005.zip › S5 File. Image data underlying Figs 5I-P/Abeta intracell tg LV-control.tif]

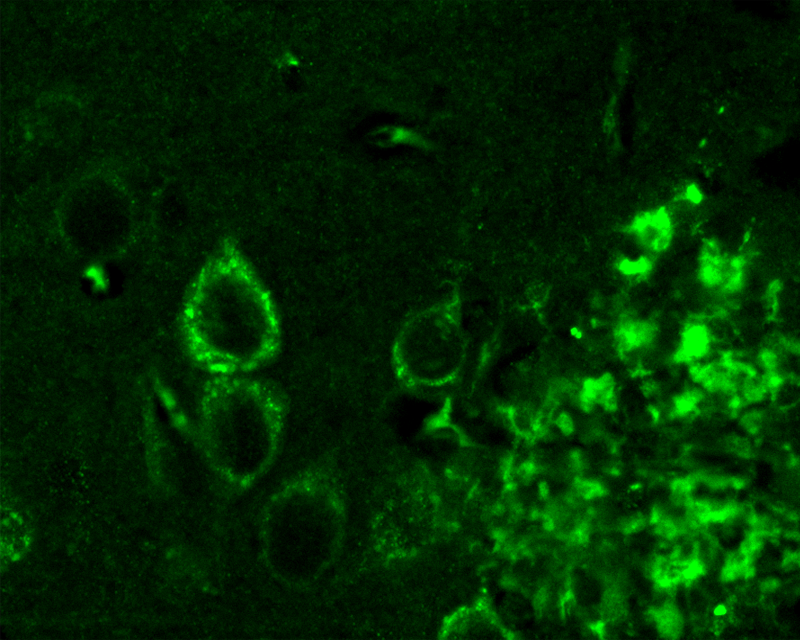

Supplement: S5 File — (ZIP) [file pone.0330647.s005.zip › S5 File. Image data underlying Figs 5I-P/Abeta intracell tg LV-NEP.tif]

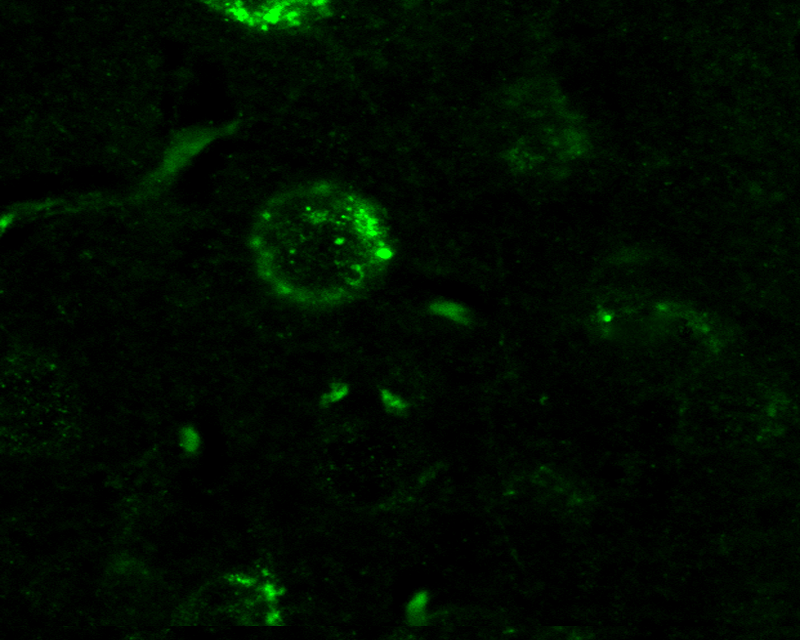

Supplement: S5 File — (ZIP) [file pone.0330647.s005.zip › S5 File. Image data underlying Figs 5I-P/Abeta intracell tg LV-secNEP apoB.tif]

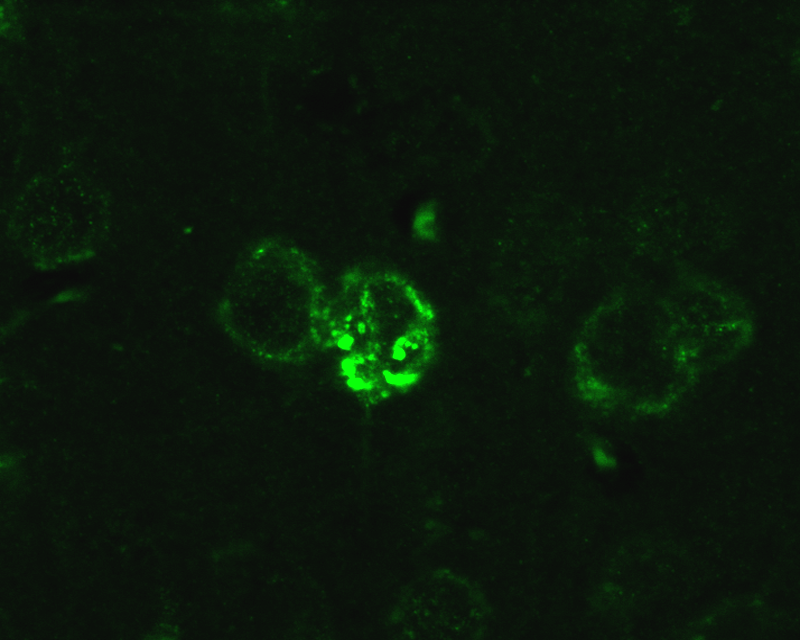

Supplement: S5 File — (ZIP) [file pone.0330647.s005.zip › S5 File. Image data underlying Figs 5I-P/Abeta intracell tg LV-secNEP.tif]

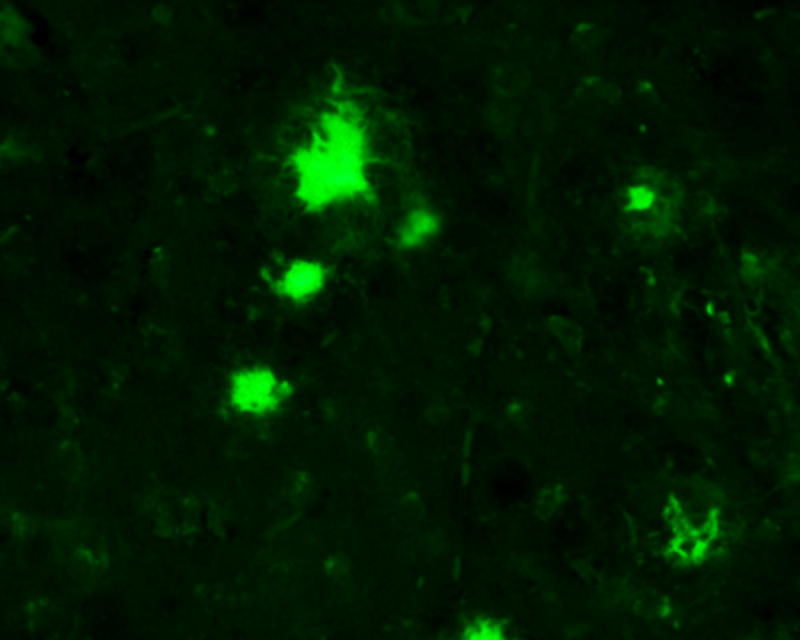

Supplement: S5 File — (ZIP) [file pone.0330647.s005.zip › S5 File. Image data underlying Figs 5I-P/Abeta plaques tg LV-control.tif]

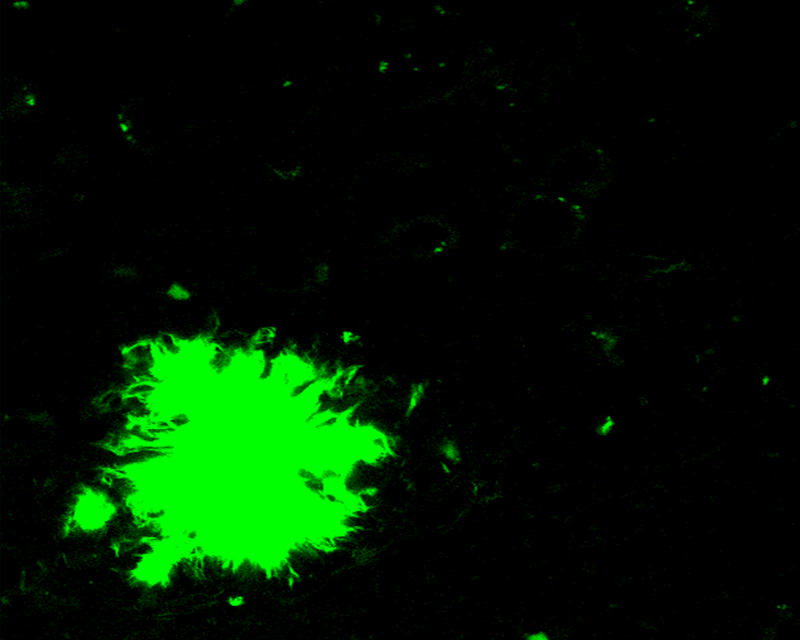

Supplement: S5 File — (ZIP) [file pone.0330647.s005.zip › S5 File. Image data underlying Figs 5I-P/Abeta plaques tg LV-Nep.tif]

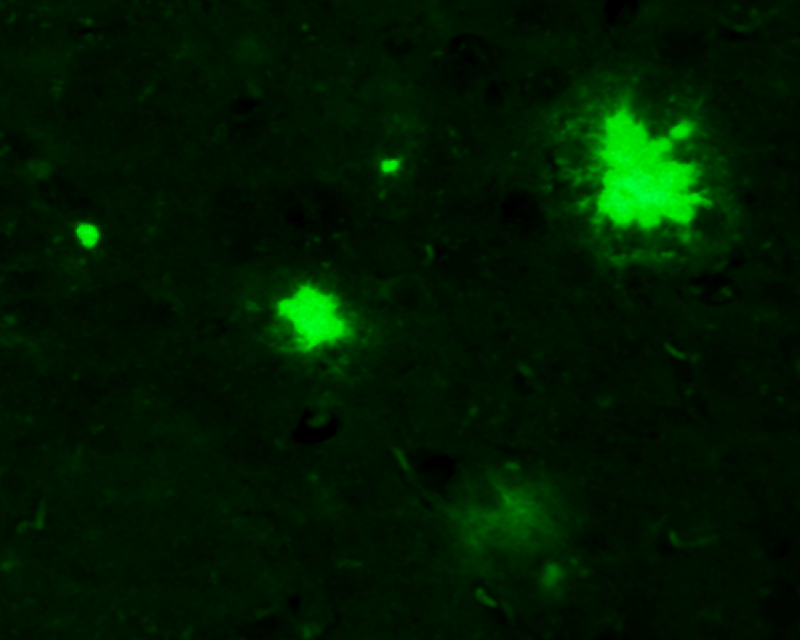

Supplement: S5 File — (ZIP) [file pone.0330647.s005.zip › S5 File. Image data underlying Figs 5I-P/Abeta plaques tg LV-sec-Nep.tif]

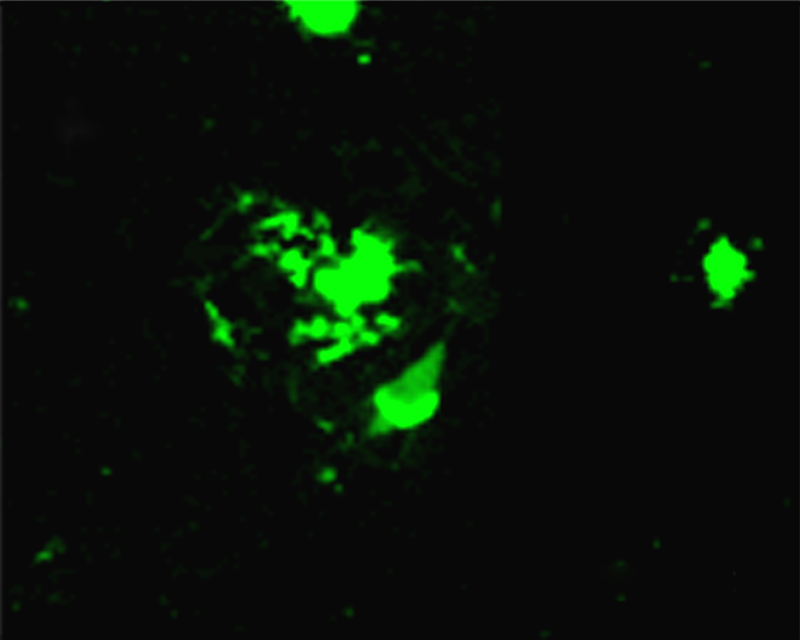

Supplement: S5 File — (ZIP) [file pone.0330647.s005.zip › S5 File. Image data underlying Figs 5I-P/Abeta plaques tg LV-secNep Apob.tif]

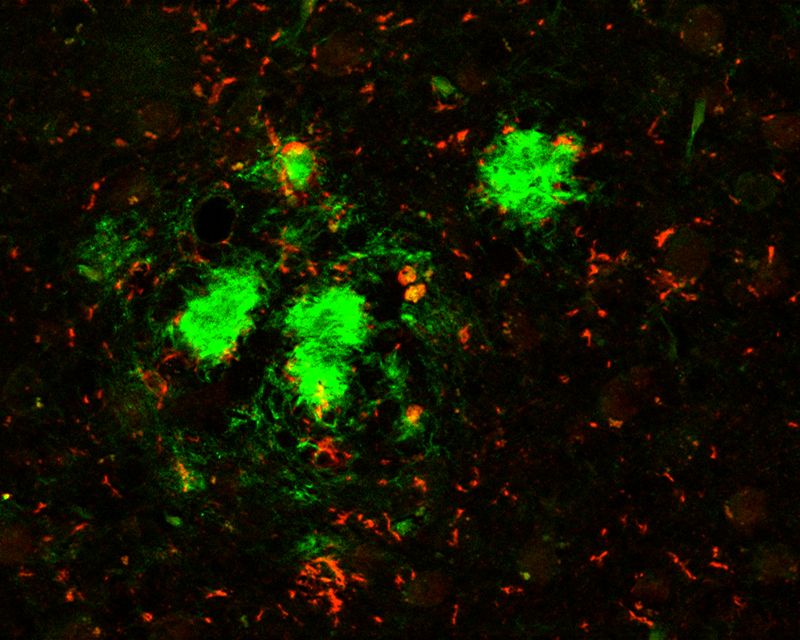

Supplement: S7 File — (ZIP) [file pone.0330647.s007.zip › S7 File - Image data underlying Figs 6C, 6F, 6G,/Apobsecnep Abeta CD68.tif]

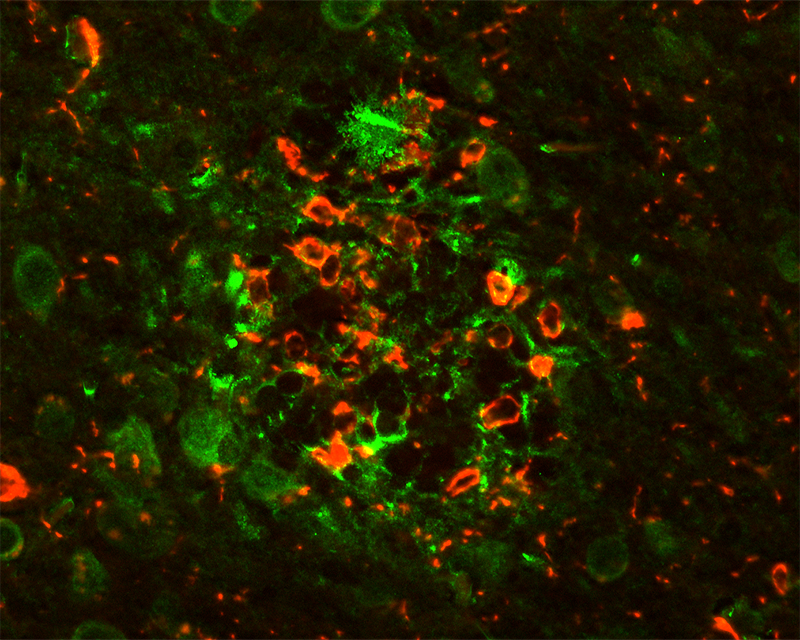

Supplement: S7 File — (ZIP) [file pone.0330647.s007.zip › S7 File - Image data underlying Figs 6C, 6F, 6G,/Apobsecnep Abeta Nep LV-apoBsec nep.tif]

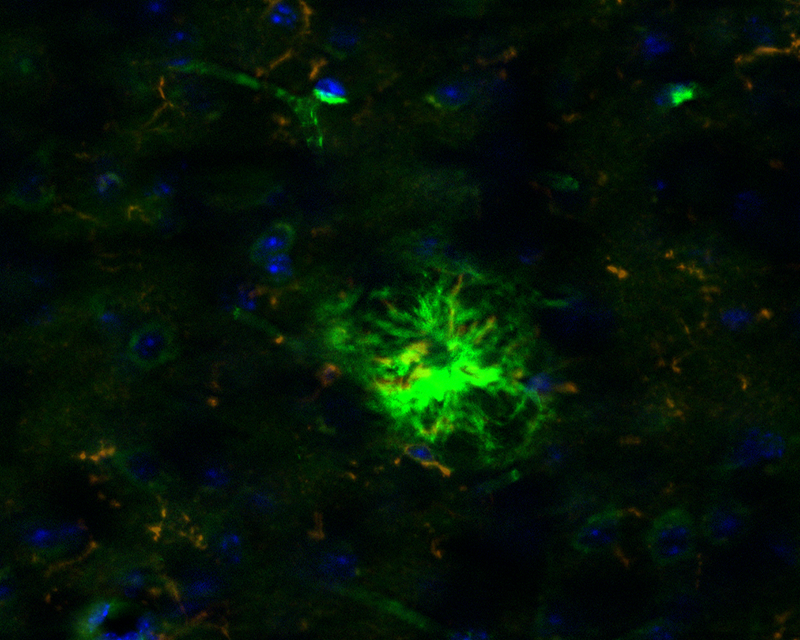

Supplement: S7 File — (ZIP) [file pone.0330647.s007.zip › S7 File - Image data underlying Figs 6C, 6F, 6G,/Apobsecnep Abeta Nep LV-ctl.tif]

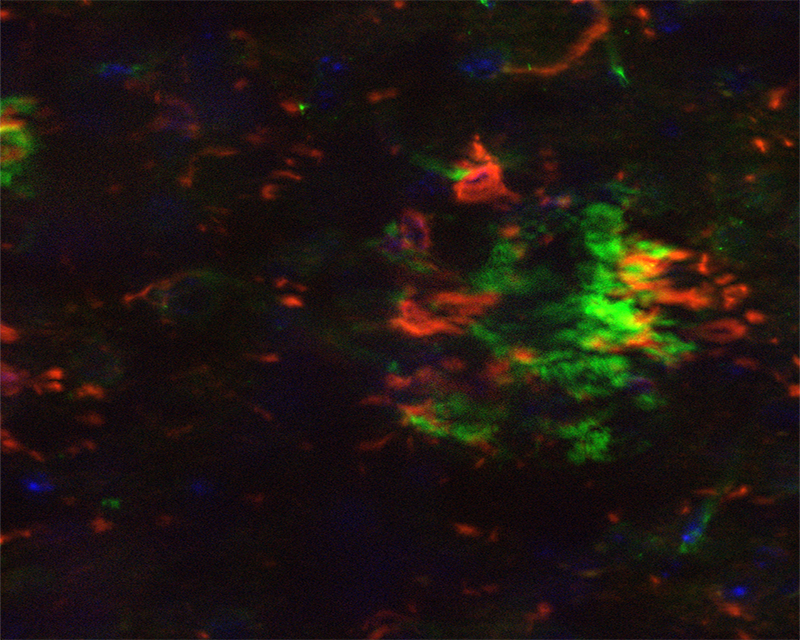

Supplement: S8 File — (ZIP) [file pone.0330647.s008.zip › S8 File. Image data underlying Figs S4C, S4F, S4I, S4L/apobsecnep Abeta CD11b LV-apobsecnep.tif]

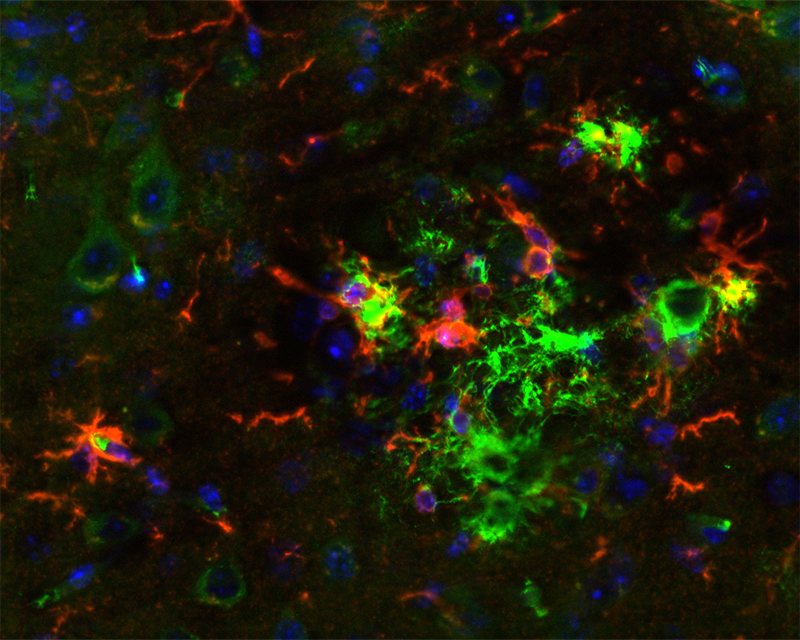

Supplement: S8 File — (ZIP) [file pone.0330647.s008.zip › S8 File. Image data underlying Figs S4C, S4F, S4I, S4L/apobsecnep Abeta CD11b LV-control.tif]

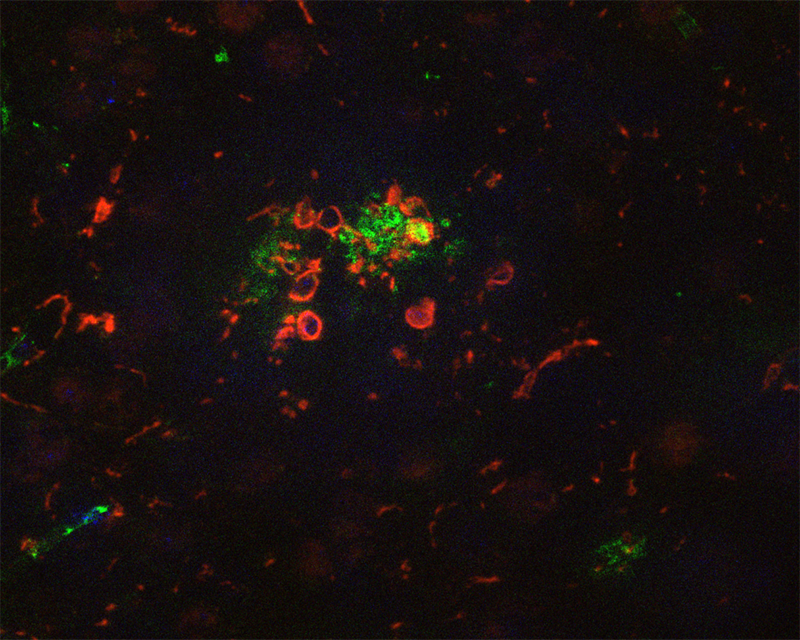

Supplement: S8 File — (ZIP) [file pone.0330647.s008.zip › S8 File. Image data underlying Figs S4C, S4F, S4I, S4L/apobsecnep Abeta iba1 LV-apobsecnep.tif]

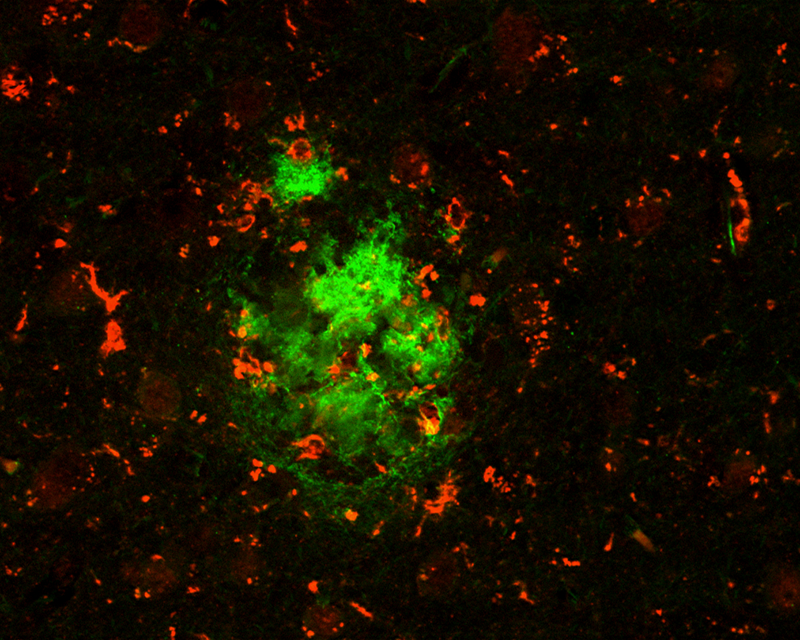

Supplement: S8 File — (ZIP) [file pone.0330647.s008.zip › S8 File. Image data underlying Figs S4C, S4F, S4I, S4L/apobsecnep Abeta Iba1 LV-control2.tif]
